# Scaling law for excitons in 2D perovskite quantum wells

**DOI:** 10.1038/s41467-018-04659-x

**Published:** 2018-06-08

**Authors:** J.-C. Blancon, A. V. Stier, H. Tsai, W. Nie, C. C. Stoumpos, B. Traoré, L. Pedesseau, M. Kepenekian, F. Katsutani, G. T. Noe, J. Kono, S. Tretiak, S. A. Crooker, C. Katan, M. G. Kanatzidis, J. J. Crochet, J. Even, A. D. Mohite

**Affiliations:** 1 Los Alamos National Laboratory, Los Alamos, NM 87545 USA; 2 Department of Materials Science and Nanoengineering, Rice University, Houston, TX 77005 USA; 3 Department of Chemistry, Northwestern University, Evanston, IL 60208 USA; 4 Univ Rennes, ENSCR, INSA Rennes, CNRS, ISCR (Institut des Sciences Chimiques de Rennes)–UMR 6226, F-35000 Rennes, France; 5 Univ Rennes, INSA Rennes, CNRS, Institut FOTON–UMR 6082, F-35000 Rennes, France; 6 Department of Electrical and Computer Engineering, Rice University, Houston, TX 77005 USA; 7 Department of Physics and Astronomy, Rice University, Houston, TX 77005 USA; 8 Department of Materials Science and Engineering, Northwestern University, Evanston, IL 60208 USA; 9 Department of Chemical and Biomolecular Engineering, Rice University, Houston, TX 77005 USA

## Abstract

Ruddlesden–Popper halide perovskites are 2D solution-processed quantum wells with a general formula A_2_A’_*n*-1_M_*n*_X_3*n*+1_, where optoelectronic properties can be tuned by varying the perovskite layer thickness (*n*-value), and have recently emerged as efficient semiconductors with technologically relevant stability. However, fundamental questions concerning the nature of optical resonances (excitons or free carriers) and the exciton reduced mass, and their scaling with quantum well thickness, which are critical for designing efficient optoelectronic devices, remain unresolved. Here, using optical spectroscopy and 60-Tesla magneto-absorption supported by modeling, we unambiguously demonstrate that the optical resonances arise from tightly bound excitons with both exciton reduced masses and binding energies decreasing, respectively, from 0.221 *m*_0_ to 0.186 *m*_0_ and from 470 meV to 125 meV with increasing thickness from *n* equals 1 to 5. Based on this study we propose a general scaling law to determine the binding energy of excitons in perovskite quantum wells of any layer thickness.

## Abstract (web summary)

Hybrid 2D layered perovskites are solution-processed quantum wells whose optoelectronic properties are tunable by varying the thickness of the inorganic slab. Here Blancon et al. work out a general behavior for dependence of the excitonic properties in layered 2D perovskites.

## Introduction

Ruddlesden–Popper halide perovskites^1,2^ (RPPs) are solution-processed quantum well structures formed by two-dimensional (2D) layers of halide perovskite semiconductors separated by bulky organic spacer layers, whose stoichiometric ratios are defined by the general formula^3^ A_2_A’_*n*-1_M_*n*_X_3*n*+1_ where A, A’ are cations, M is a metal, X is a halide and the integer value *n* determines the perovskite layer thickness (or quantum well thickness). Recent breakthrough in the synthesis of phase-pure (a single *n*-value) RPPs with higher values^3–5^ of *n*, up to *n* equals to 5, has inspired their use as low-cost semiconductors in optoelectronics^5–8^ as an alternative to three-dimensional (3D) perovskites due to their technologically relevant intrinsic photo- and chemical-stability^5–10^. However, key fundamental questions remain unanswered in RPPs with *n* greater than 1, such as the nature of optical transitions, as well as the behavior of Coulomb interactions especially with increasing quantum well thickness. In fact there has been an intense ongoing debate^6–8,11–13^ regarding the exact nature of the optical transitions (excitons versus free carriers) in RPPs with large *n*-values. RPPs with *n* equals to 1 (excitons at room temperature),^14,15^ and 3D perovskites^16^ (free carriers at room temperature) are representative of the two limiting regimes at room temperature, but the analysis of the crossover has not been performed. This issue originates from the lack of knowledge of the fundamental quantities such as the exciton reduced mass, dielectric constant and characteristics like the spatial extension of electron and hole wavefunctions, which play a crucial role in the determination of the exciton binding energy. In particular, contradictory reports of the value of the exciton reduced mass have significantly contributed to this uncertainty^15,16^ making it the most critical experimentally derived parameter required for the quantitative determination of the exciton characteristics in hybrid (organic–inorganic) perovskite systems. Moreover, unlike bulk inorganic semiconductors, heterostructures^17^ and 3D perovskites^18,19^, it is non-trivial to determine the exciton reduced mass in layered hybrid perovskites using a symmetry-based (**k.p**) approach^20^ or using many-body ab initio calculations^21^. Furthermore, ambiguity in the determination of the exciton binding energy in layered 2D perovskites has also been imposed by the limited understanding of the role of dielectric confinement versus quantum confinement with increasing quantum well thickness^22,23^. More generally, phase-pure RPPs with *n* greater than 1 present a unique opportunity to explore the physical properties of natural quantum well semiconducting crystals intermediate between monolayer 2D materials^24,25^ and 3D materials^26^, a quasi-dimensional physics only accessible in synthetic inorganic semiconductor quantum well structures so far^26^.

**Fig. 1:**
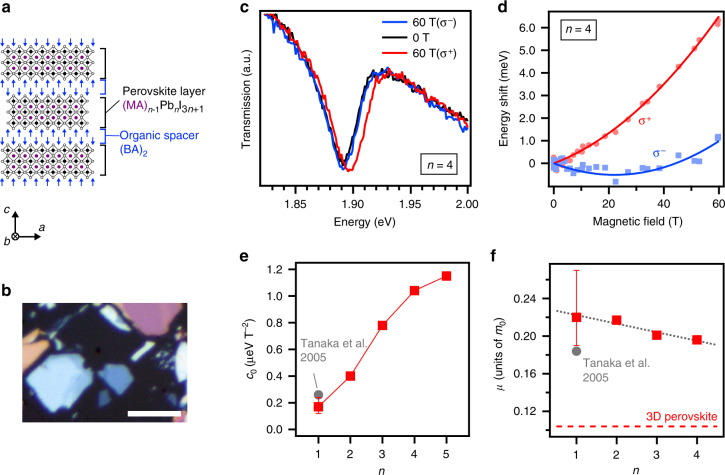
Exciton reduced mass from magneto-absorption spectroscopy and theory. **a** Schematic of the RPP structure cut along the direction ĉ of stacking of the 2D layers. **b** Image of mechanically exfoliated RPP crystals. Scale bar is 10 µm. **c** Magnetic field dependence of the light transmission of an individual RPP with *n* equals to 4 crystal for right- (σ^‒^) and left-handed (σ^+^) circular polarization. **d** Corresponding shift of the exciton energy as a function of the magnetic field. Fit of the data using Δ*E* = ±1/2 *g*_0_*μ*_B_*B* + *c*_0_*B*^2^ yields *c*_0_ = 1.04 ± 0.16 µeV T^−2^ and *g*_0_ = 1.59 ± 0.03. **e** Derived diamagnetic shift coefficient of the measured RPPs (red squares). The value from Tanaka et al.^15^ is also reported for RPPs with *n* equals to 1 with a slightly larger organic spacer. **f** Exciton reduced mass derived from fitting the diamagnetic shifts with our theoretical model. The gray dotted line is a guide for the eyes. The red dashed line indicates average value of exciton reduced mass for the 3D perovskite MAPbI_3_. Error bars correspond to s.d. from the fit of the shift of the exciton energy as a function of the magnetic field

Here we present the study on using low-temperature (4 K) magneto-optical spectroscopy to accurately determine the exciton reduced mass for (BA)_2_(MA)_*n*-1_Pb_*n*_I_3*n*+1_ RPP crystals with perovskite layer thickness varying between 0.641 and 3.139 nm, corresponding to *n* varying between 1 and 5 (Fig. 1a, Supplementary Fig. 1, and Supplementary Table 1), where BA and MA stand for CH_3_(CH_2_)_3_NH_3_ and CH_3_NH_3_, respectively. The reduced mass is used to develop a generalized theoretical model for electron–hole interactions in RPPs and determine fundamental characteristics of the exciton states. In parallel, from low-temperature optical spectroscopy we experimentally determine the exciton binding energy in the RPPs with *n* varying from 1 to 5. Finally, from these results we produce a general scaling behavior for the binding energy of Wannier–Mott exciton states in RPPs, which allows for prediction of the exciton binding energy for any given thickness. This study closes a long-standing scientific gap and will lead to the rational design of next-generation layered 2D perovskite-based optoelectronic devices.

## Results

### Exciton reduced mass from magneto-absorption measurements

Fig. 1 shows the RPP structure and results from magneto-absorption spectroscopy, which was employed to probe the strength of the electron–hole interaction in RPPs and deduce the exciton reduced mass. These measurements were performed at 4 K in the Faraday geometry (magnetic field along the stacking direction of the layers, **ĉ**-axis), and the optical spectra of the right- and left-handed circular polarization components (σ^±^) of the transmitted white light were probed. Application of a high magnetic field on the RPP with *n* equals to 4 results in an energy shift of the optical resonance at about 1.9 eV (Fig. 1c, d). Similar results were obtained for the other RPPs (see Supplementary Fig. 2). This optical transition was identified as the exciton ground state and its energy shift under magnetic field in the Faraday configuration is expressed by^26^ Δ*E* = ±1/2 *g*_0_*μ*_B_*B* + *c*_0_*B*^2^, where the first term describes the Zeeman splitting of the σ^+^ and σ^‒^ exciton transitions (*g*_0_ is the *g*-factor in the perovskite plane, *μ*_B_ the Bohr magneton, *B* the magnetic field) and the second one the diamagnetic shift (*c*_0_ is the diamagnetic shift coefficient). Under magnetic field the competition between the Zeeman and diamagnetic effects explains the non-symmetric, opposite-sign energy shift of the σ^+^ and σ^‒^ exciton transitions with respect to the zero field exciton absorption energy (Fig. 1d and Supplementary Fig. 2). We note that this asymmetry becomes more pronounced for thicker perovskite layers (*n* approaching 5), which is a consequence of the strong increase of the diamagnetic coefficient with increasing *n*-value. Fitting the exciton energy shifts for both σ^+^ and σ^‒^ polarizations using the model above yields the diamagnetic coefficients *c*_0_ of the RPPs (Fig. 1e). Here, *c*_0_ increases monotonically with the perovskite layer thickness, and it ranges from ~0.2 to ~1.1 µeV T^−2^ for the RPPs with *n* varying from 1 to 5, respectively. We note that due to relatively small signal-to-noise ratio in the magneto-absorption data for the RPP with *n* equals to 1 (Supplementary Fig. 2a), the diamagnetic coefficient for this compound was obtained from a combined experimental–theoretical approach which consisted in fitting the *c*_0_ value to be in best agreement with both the magneto-absorption data and the experimental exciton binding energy discussed later in this paper. For comparison, we also included in Fig. 1e the value of *c*_0_ reported by Tanaka et al.^15^ (*c*_0_ = 0.26 µeV T^−2^) for a RPP with *n* equals to 1 and with a slightly larger organic spacer (hexyl-ammonium CH_3_(CH_2_)_5_NH_3_ instead of butylammonium CH_3_(CH_2_)_3_NH_3_ in our case). We note that our value of *c*_0_ for the RPP with *n* equals to 1 with BA as organic spacer is in agreement but slightly smaller than other reports of RPPs with *n* equals to 1 with longer organic spacers^27–29^. Concomitant with the diamagnetic shift increase with *n*-value, the *g*-factor presents a marked increase from 0.8 to 1.6 for the RPPs with *n* varying from 1 to 5, and a value of about 1.7 was derived from our 3D perovskites magneto-absorption data (Supplementary Fig. 3) and is consistent with the literature^30,31^.

The diamagnetic coefficient is directly connected to both the exciton reduced mass and the strength of the electron–hole Coulomb interaction. Although a simple relation exists for pure 2D systems where quantum confinement dominates^22,26^, i.e., when confinement effects stem from the confinement of electron and hole wavefunctions in a 2D plane, this model does not apply to the RPPs due to the following reasons. First, the perovskite layer thickness is comparable to the spatial extent of the excitons^15,16,27^ and the exciton wavefunction cannot be strictly confined to a 2D plane (see also Supplementary Note 1 to 4). Second, the dielectric confinement plays a key role in the photo-physics of RPPs^15,23,27^. Dielectric confinement (also called image charge effect) manifests in a quantum well system provided that the thickness of the quantum wells is comparable to the exciton size, and the dielectric constant ratio between the quantum wells (perovskite layers with *ε*_w_~4 or higher) and the barriers (organic spacing layers with *ε*_b_~2.2) is larger than unity. This result leads to an enhancement of the Coulomb interaction between the electron and hole pair composing each exciton, which is a consequence of the reduced dielectric screening of the exciton electric field partially located outside the quantum well, i.e., in the surrounding barriers that have a lower dielectric constant.

Therefore, we developed a theoretical model (see details in the next section, Supplementary Note 1, and Supplementary Fig. 4), which describes the electron–hole Coulomb interaction in thin semiconductors and includes dielectric confinement (Keldysh theory^22^). For this theoretical model to work, it requires an accurate determination of the exciton reduced mass (labeled *µ*). We evaluated the reduced mass for each RPP by adjusting the theoretical values of *c*_0_ to those measured experimentally (Fig. 1f and Supplementary Note 5). This yielded *µ* = 0.221 *m*_0_ (*m*_0_ is the free electron mass) for the RPP with *n* equals to 1, and 0.184 *m*_0_ for the RPP with *n* equals to 1 compound with longer organic spacers reported by Tanaka et al.^15^. Then, we observe a monotonic decrease of the exciton reduced mass from 0.217 *m*_0_ for the RPP with *n* equals to 2 down to 0.196 *m*_0_ for the RPP with *n* equals to 4. Due to computational limitations, our model was not applied to the RPP with *n* equals to 5; however we estimated, to a first approximation, *µ* = 0.186 *m*_0_ from the extrapolation of the data for the RPPs with *n* equals 1 to 4.

The deduced values of exciton reduced mass in the RPPs up to *n* equals to 5 are significantly larger than those reported for 3D perovskites (Supplementary Fig. 3), even though the exciton states are well confined within the perovskite layer (see next section). This is surprising because a decrease of the exciton reduced mass is expected for increasing perovskite layer thickness, with limiting value of 0.104 *m*_0_ (3D perovskite), as the vicinity of the exciton approaches that of the 3D perovskites. However, the equivalent quantum well is still very thin even for the RPP with *n* equals to 5. Moreover, the reduced mass of the exciton ground state calculated by density functional theory (DFT) for each RPP (with *n* equals 1 to 4) yields a similar dependence on the *n*-value as the experimental ones provided the underestimation of the bandgap computation by DFT is taken into account (Supplementary Fig. 5). We explain the higher values of exciton reduced mass in the RPPs studied as compared to their 3D counterpart by a progressive reduction of the energy bandgap^26^. The limiting 3D value is thus expected to be reached for larger *n*-value, i.e., when the RPP bandgap equals the 3D perovskite one. Furthermore, electronic band mixing and non-parabolicity effects have been known to induce changes in the exciton reduced mass in quantum well systems^32–35^. These effects have also been discussed in classic inorganic semiconductors and agree with the *n*-value dependence of the experimental bandgap, diamagnetic shift and *g*-factor (Figs. 1e and 3a, and Supplementary Fig. 6). In fact, the bandgap dependence of the exciton reduced mass was also reported recently in 3D hybrid perovskites^31^. To conclude, the unambiguous determination of the reduced mass allows us to develop a predictive model for excitons in solution-processed quantum well systems and calculate the exciton binding energies of the RPP compounds.

### Calculating the exciton binding energy in 2D perovskites

**Fig. 2:**
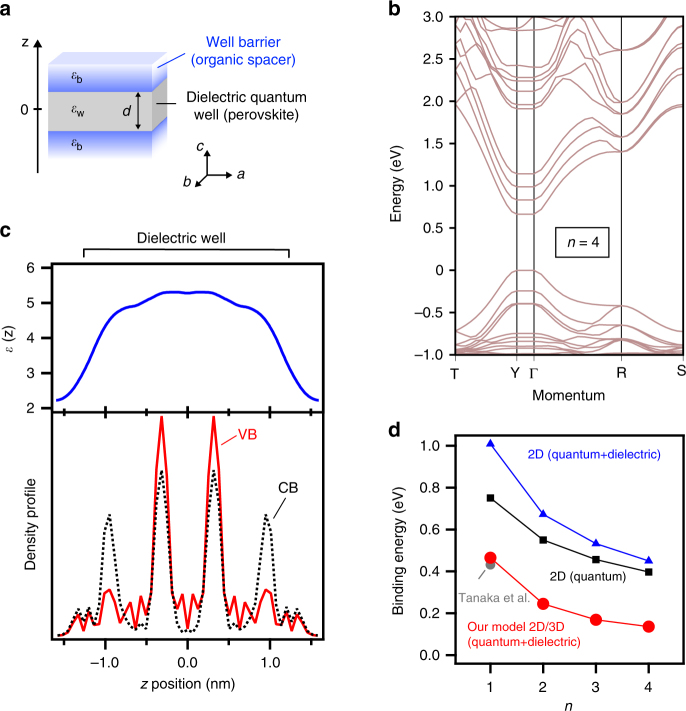
Semi-empirical model of Wannier–Mott exciton in RPPs. **a** Schematics of the single quantum well system to which our model was applied to. **b** Computed band structure for the RPP with *n* equals to 4. **c** Corresponding electron (dashed black lines) and hole (red) probability density profiles, and high-frequency dielectric constant profile (blue) along the stacking axis **ĉ**. **d** Calculated binding energy of the exciton ground state using the exciton reduced mass in Fig. 1f and the DFT results. The values calculated using our model results (red circles) is compared to those obtained in the approximation of a pure 2D system (blue triangles) and of a pure 2D system without dielectric confinement (black squares). The binding energy of the excited exciton states (2*s*, 3*s*, 4*s*) were also computed and are reported in Supplementary Fig. 7. For reference, the 3D perovskite MAPbI_3_ yields an exciton binding energy of about 16 meV, from Miyata et al.^16^ and in agreement with our results in Supplementary Fig. 3

The binding energy of the exciton ground state was calculated for each RPP (with *n* equals 1 to 4) by inputting each respective value of the exciton reduced mass into our theoretical model and semi-empirically solving the Bethe–Salpeter equation based on the effective mass Green’s function approach^23^. This approach includes: calculating the electronic structures of the RPPs to extract exciton wavefunctions and dielectric constant profiles using DFT, generalizing the Keldysh theory for 2D perovskites and combining both of the above by building a semi-empirical model to simulate the Wannier–Mott exciton characteristics (Fig. 2 and Supplementary Note 1, 2, and 6). In our model, the potential function describing the Coulomb interaction between the electron and hole forming the exciton states is based on Keldysh theory^22^ generalized to a semiconducting dielectric quantum well (Fig. 2a), i.e., a dielectric well (perovskite layer) with thickness *d* and dielectric constant *ε*_w_ sandwiched between well barriers (organic spacing layers) with dielectric constant *ε*_b_. The non-local, screened electron–hole pair interaction potential is given by:

1 $$\begin{array}{c}V_{\mathrm{s}}\left( {q_{\mathrm{t}}} \right) = \frac{{ - e^2}}{{2\varepsilon _{\mathrm{w}}q_{\mathrm{t}}}}{\displaystyle\int} \!\!\!{\mathop {\displaystyle\int}_{\!\!\!\!{\frac{{ - d}}{2},\frac{{ - d}}{2}}}^{\frac{d}{2},\frac{d}{2}} \;{\rho _{\mathrm{e}}\left( {z_{\mathrm{e}}} \right)\rho _{\mathrm{h}}\left( {z_{\mathrm{h}}} \right)\left[ {e^{ - q_{\mathrm{t}}\left| {z_{\mathrm{e}} - z_{\mathrm{h}}} \right|}} \right.} } \\ \qquad + {\mathrm{\Delta }}\chi \left( {e^{ - q_{\mathrm{t}}\left| {z_{\mathrm{e}} + z_{\mathrm{h}} - d} \right|} + e^{ - q_{\mathrm{t}}\left| {z_{\mathrm{e}} + z_{\mathrm{h}} + d} \right|}} \right)\\ \qquad \qquad \qquad \left. { + {\mathrm{\Delta }}\chi ^2\left( {e^{ - q_{\mathrm{t}}|z_{\mathrm{e}} - z_{\mathrm{h}} - 2d|} + e^{ - q_{\mathrm{t}}|z_{\mathrm{h}} - z_{\mathrm{e}} - 2d|}} \right)} \right]\mathrm{d}z_{\mathrm{e}}\mathrm{d}z_{\mathrm{h}},\end{array}$$

in reciprocal space and in the in-plane $\left( {{\hat{\mathbf a,}}{\hat{\mathbf b}}} \right)$ direction. In Eq. (1), the difference of electron and hole transverse (in-plane) wavevectors is *q*_t_, ${\mathrm{\Delta }} = \left( {1 - \chi ^2e^{ - 2q_{\mathrm{t}}d}} \right)^{ - 1}$, and $\chi = \frac{{\varepsilon _{\mathrm{w}} - \varepsilon _{\mathrm{b}}}}{{\varepsilon _{\mathrm{w}} + \varepsilon _{\mathrm{b}}}}$. Here, the dielectric constants are inputs from our DFT results and were taken at optical frequency because the values of binding energy measured in RPPs (see next section) are one order of magnitude larger than the highest energy value reported for the lattice optical phonon modes^36^. The electron (*ρ*_e_) and hole (*ρ*_h_) probability density profiles along the stacking axis **ĉ** (*z*_e,h_ are real-space coordinates along this direction) were also obtained from our DFT results. This method is equivalent to the real-space computation of the screening effect using the image charge method^37^. We also note that the general expression (1) of the electron–hole interaction potential corresponds to the general Keldysh potential in real space^22^. Upon different degrees of approximation (as discussed in Supplementary Note 3), the electron–hole interaction potential used in the literature in the limit of monolayer 2D transition metal dichalcogenides^38–42^ can be retrieved. However, these approximations are not valid in RPPs (Supplementary Note 4) and the general expression (1) needs to be used.

Overall, theoretical solution to the exciton binding energy in each RPP requires the knowledge of: the electron and hole probability density distributions, the dielectric constant profile along the stacking direction, and the exciton reduced mass. In RPPs, the density profiles and dielectric constants along the stacking direction (Fig. 2c and Supplementary Fig. 4b), along with the electronic band structure (Fig. 2b), were derived from the electron and hole wavefunctions calculated by DFT (Supplementary Note 6). A representative example of the electron and hole probability density profiles is sketched in Fig. 2c for the RPPs with *n* equals to 4. Our calculations reveal that the charge wavefunctions stay confined into the perovskite layers and exhibit little leakage into the organic spacing layers, which is consistent with uncoupled quantum well systems. Moreover, the results reveal maximum values of dielectric constant in the range of 4 to 5.2 for the perovskite layers in RPPs with *n* equals 1 to 5, and a minimum value of about 2.2 for the organic spacing layers, consistent with previous estimations^43,44^. The contrast of dielectric constants between the perovskite and organic spacers is at the origin of the strong dielectric confinement effects observed in RPPs, as discussed in the next section.

Gathering all these information, our general model was then applied to the calculation of the exciton binding energy in RPPs, yielding a value of 467 meV for the RPP with *n* equals to 1 (435 meV for the case of longer organic spacers from Tanaka et al.^15^), and which decreases monotonically to ~135 meV for the RPP with *n* equals to 4 as illustrated in Fig. 2d (see also Supplementary Fig. 7 and Supplementary Table 2). We observe a radical difference of the exciton binding energies calculated using our general approach as compared to those obtained under various degrees of approximation usually suited to 2D materials such as transition metal dichalcogenides^38–42^, i.e., in the case the charges are strictly restricted to a 2D plane and including or not dielectric confinement (see details in Supplementary Note 3). The accuracy of our model to predict the binding energies of excitons in 2D perovskites was then verified by directly measuring these values using optical spectroscopy techniques.

### Direct measurement of the exciton binding energy

**Fig. 3:**
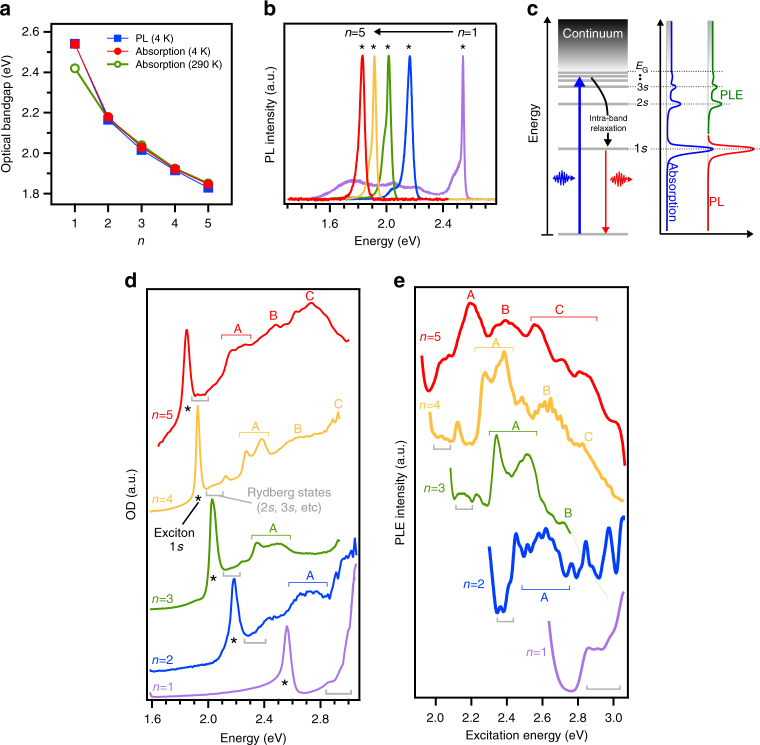
Optical spectroscopy of the RPP crystals with *n* equals 1 to 5. **a** Experimental optical bandgap scaling. **b** Photoluminescence spectra. **c** Schematics of the Rydberg series of the exciton ground state (1*s*) and excited exciton states (2*s*, 3*s*, etc.) merging with the continuum. (Right) Corresponding absorption or optical density, photoluminescence PL, and photoluminescence excitation PLE spectra typically observed in 2D material systems^26,49^. **d** Optical density OD and **e** PLE spectra of the RPPs with *n* equals 1 to 5, respectively. Stars point to exciton ground state optical transitions, black squares show the continuum onsets and gray brackets indicate the region of excited exciton states. Labels A, B and C indicate absorption regions at energies higher than the continuum bandgap *E*_G_ and which are apparently common to all the RPPs given an energy shift proportional to the bandgap

We directly measured the exciton binding energy in each RPP using optical absorption, photoluminescence (PL) and photoluminescence excitation (PLE) spectroscopy (Fig. 3). The optical bandgap was tuned over the visible spectral range from 2.540 ± 0.004 eV (488 nm) in the RPP with *n* equals to 1 down to 1.846 ± 0.004 eV (672 nm) in the RPP with *n* equals to 5 (Fig. 3a). We note that the RPPs with *n* greater than 1 retained their optical bandgap from 290 K down to 4 K. This observation implies that our study conducted at low temperature is directly transposable to room temperature and provides direct insights into the photo-physics of materials used in practical devices.

Figure 3b shows a single sharp peak in the PL spectra, indicating the emission of the exciton ground state^14,45^. The low-energy shoulder observed more than tens of meV below the exciton peak in all RPPs was recently assigned in the RPP with *n* equals to 1 to a phonon replica^46^ and self-trapped excitons^47^. Based on power dependence measurements of the PL (Supplementary Fig. 8), we hypothesize a similar origin for the resonances in the RPPs with *n* greater than 1, but we only focus on investigating the ground state exciton transition in this study.

All the presented absorption spectra show similar features (Fig. 3d), which includes a single absorption peak at low energy corresponding to the exciton ground state resonance. Moreover, a steady increase of absorption at higher energies modulated by energy optical transitions is well reproduced in the PLE measurements (Fig. 3e). These spectral features were assigned to the Rydberg series of the Wannier–Mott exciton (1*s*, 2*s*, 3*s*, 4*s* noted as *Ns*, with *N* equals to 1, 2, 3 and 4 and having energies *E*_*Ns*_) and are illustrated in Fig. 3c. The onset of the continuum *E*_G_ corresponds to electron and hole free carrier states (Fig. 3c), as previously reported in the RPP with *n* equals to 1 perovskites^15,45^, other 2D nanomaterials^39,48^ and quantum well semiconductors^26,49^. Absorption transition features, marked as A, B and C, are observed in both the absorption and PLE spectra for energy higher than E_G_, and are apparently common to all the RPPs given an energy scaling with the *n*-value proportional to the bandgap scaling (Fig. 3d, e). Close to the continuum onset and in our experimental energy range, the optical transitions in RPPs at energies above *E*_G_ have been assigned^11,14,50^ to transitions between the Pb(6*s*)-I(5*p*) states in the valence band and Pb(6*p*) states in the conduction band^20,51^. The absorption features A, B and C can be understood as transitions between valence and conduction bands overlapping with the continuum of states from the band-edge exciton. For example, from the calculated RPP band structures (Fig. 2b and Supplementary Fig. 4a), one can assume that the high energy optical transitions involve the series of the mini-bands directly below the highest valence band and above the lowest conduction band at the Γ high symmetry point in the Brillouin zone. Calculation of the broadband optical spectra in the RPPs, including dipole allowed transitions from group theory and joint density of states, should be performed in future theoretical studies in order to understand the details of the experimental absorption spectra at energies higher than the continuum *E*_G_.^20,51^

**Fig. 4:**
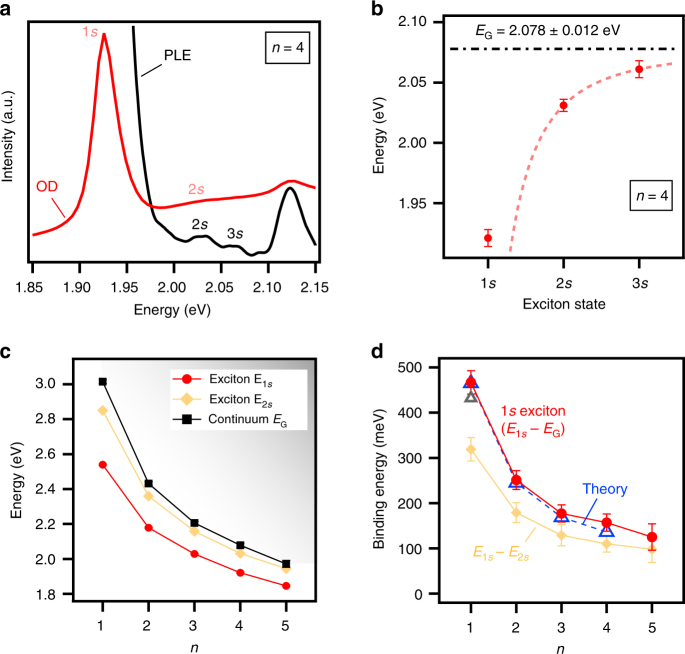
Direct measurement of the exciton binding energy and Rydberg states in RPPs with *n* equals 1 to 5. **a** Optical density OD and photoluminescence excitation PLE spectra of the RPP with *n* equals to 4 (see the others in Supplementary Fig. 9) clearly showing the exciton ground state 1*s* and the excited exciton states 2*s* and 3*s*. **b** Corresponding energy of the exciton Rydberg states. Dashed line is a fit to the 2*s* and 3*s* states with the 2D hydrogen model^26^ of exciton Rydberg series using *R*_y_ = 0.11 ± 0.04 eV and *E*_G_ = 2.078 ± 0.012 eV in the formula *E*_*Ns*_ = *E*_G_ − *R*_y_ /(*N* − 1/2)^2^, with *Ns* equals to 1*s*, 2*s*, 3*s*. **c** Evolution of the exciton ground state, first excited state 2*s*, and continuum energies with the 2D perovskite layer thickness (or *n*). **d** Corresponding experimental binding energy of the exciton ground state (1*s*) and the excited exciton states (2*s*, 3*s*, 4*s*), and comparison to theoretical results for the exciton ground state. The gray open triangle corresponds to the 1*s* exciton binding energy calculated for the diamagnetic shift obtained by Tanaka et al.^15^ in a RPP with *n* equals to 1 with larger organic spacers (see Fig. 1d, e). The values *E*_1*s*_ − *E*_2*s*_ provide a lower limit for the exciton binding energy. Error bars correspond to s.d. from the determination of the energy of the exciton features

The exciton binding energies in RPPs were derived from the study of the optical transitions corresponding to the exciton Rydberg series and the continuum. We present a representative example for the RPP with *n* equals to 4 in Fig. 4a, b. Dielectric confinement (or image charge effect) has been shown^15,27,44,45^ to mainly influence the 1*s* excitons in RPPs with *n* equals 1. Therefore, the 2*s* and 3*s* exciton states were fitted using the classic 2D hydrogen Rydberg series with energies^26^ *E*_*Ns*_ = *E*_G_ − *R*_y_ /(*N* − 1/2)^2^, where *R*_y_ is the Rydberg energy. This yields *E*_G_ = 2.078 ± 0.012 eV and *R*_y_ = 0.11 ± 0.04 eV for the RPP with *n* equals to 4 (Fig. 4b, dashed red line). This procedure is similar to the one used in transition metal dichalcogenides^39,52^ and 2D perovskites^15,45,53^. It was also applied to the RPPs with *n* equals to 1, 2, 3 and 5 (Supplementary Fig. 9), i.e., the 1*s*, 2*s* and 3*s* exciton states were identified from the analysis of the experimental spectra and the 2*s*, 3*s* Rydberg series were fitted with the 2D hydrogen model to derive the continuum energy *E*_G_. The binding energy of the 1*s* exciton ground state was derived from the difference |*E*_1*s*_ − *E*_G_|, which ranges from about 470 meV for the RPP with *n* equals to 1 down to 125 meV for the RPP with *n* equals to 5 (Fig. 4c, d, Supplementary Table 1). We note that the lower limit of the exciton binding energy is given by the energy difference between the (1*s*) exciton ground state and (2*s*) first excited state (Fig. 4d). We emphasize that the predicted theoretical and experimentally determined values are in excellent agreement (Fig. 4d and Supplementary Fig. 7), thus validating the accuracy of our developed theoretical model for Wannier–Mott excitons in RPPs. These results emphasize the need for an accurate determination of the exciton reduced mass and taking into account the contributions of quantum and dielectric confinements as well as hole and electron density profiles in order to develop an accurate and robust theoretical model for the 2D perovskite systems.

After validating the model, we can further extract pertinent details on the specific role of dielectric confinement on the exciton characteristics and the scaling of the exciton binding energy with perovskite layer thickness. The model elucidates that the overlap of the electron and hole wavefunctions, whose convolution forms the exciton ground state, becomes more pronounced towards the center of the perovskite layer for the highest *n*-value (Fig. 2c and Supplementary Fig. 4c). This suggests that the electric field lines for the exciton ground state are significantly more localized within the perovskite layer for larger *n*-values than for the RPP with *n* equals to 1 and 2. This is consistent with the fact that with increasing perovskite layer thickness the strength of dielectric confinement decreases with respect to quantum confinement (Fig. 2d). Furthermore, the calculated probability densities of the electron and hole wavefunctions exhibit negligible intensity outside of the perovskite layer (i.e., into the organic spacer layer) as displayed in Fig. 2c and Supplementary Fig. 4c, which is comparable to a technologically relevant multi quantum well system. In summary, our theoretical model demonstrates that the strong exciton binding energy in thin 2D perovskite layers (*n* approaching 1) stems from strong dielectric confinement effects, which wane progressively for larger perovskite layer thicknesses (*n* approaching 5 or greater).

### Simple analytical expression of the exciton binding energy scaling law

Finally, based on our understanding of the exciton behavior with varying perovskite layer thickness, we developed a general empirical scaling law of the exciton binding energies based on a classical model for low dimensional systems^54^ as described in Eq. (1):

2 $$E_{{\mathrm{b}},1{\mathrm{s}}} = \frac{{E_0}}{{\left( {1 + \frac{{\alpha - 3}}{2}} \right)^2}}{\mathrm{with}}\;{\mathrm{\alpha }} = 3 - \gamma e^{ - \frac{{L_{\mathrm{w}}}}{{2a_{0}}}}.$$

**Fig. 5:**
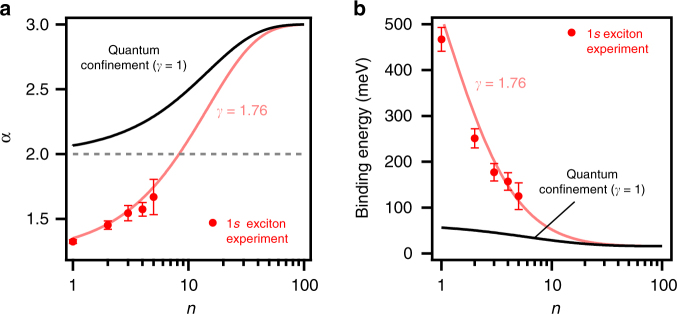
Scaling law of the exciton binding energy with the perovskite layer thickness. **a** The dimensionality coefficient *α* was derived from Eq. (2), where the exciton binding energy are the experimental values of Fig. 3d and *E*_0_ = 16 meV, *a*_0_ = 4.6 nm, and *L*_w_ = 0.6292 × *n* in nanometers.The black curve indicating *γ* equals to 1 corresponds to the case of pure quantum confinement in quantum well systems with infinite potential barriers. The red curve indicating *γ* equals to 1.76 was derived from the fit to the experimental values of *α* (red markers) using the expression of *α* in Eq. (2). Setting *γ* greater than 1 leads to a decrease of the value of *α* which reflects the more pronounced compression of the exciton ground state wavefunction in the perovskite layer due to dielectric confinement, as compared to the case of pure quantum confinement. **b** Corresponding results for the binding energy of the exciton ground state, showing the enhancement of the binding energy due to dielectric confinement. The red curve gives the general scaling law of the exciton binding energy with the perovskite layer thickness based on the Eq. (2), with *E*_0_ = 16 meV, *γ* = 1.76, *a*_0_ = 4.6 nm, and *L*_w_ = 0.6292 × *n* in nanometers. Error bars correspond to s.d. reported from the analysis in Fig. 4

In this expression for the exciton ground state binding energy, *E*_0_ (=16 meV) and *a*_0_ (=4.6 nm) are the 3D Rydberg energy and Bohr radius of 3D perovskites^16^, respectively, and *L*_w_ is the physical width of the quantum well (Supplementary Table 1) for an infinite quantum well potential barrier^55^. In the model (2), the exciton is considered isotropic in a *α*-dimensional space (*α* strictly greater than 1 and smaller than 3) and we introduce an empirical correction factor *γ*. The factor *γ* is a simple way to empirically account for the deviations from the pure quantum confinement regime, including electron and holes densities and dielectric confinement effects. In a purely quantum confined regime *γ* equals to 1 in the expression of *α*, but experimentally derived *α*−value in RPPs can only be fitted with a larger *γ* value, i.e., *γ* equals to 1.76 (Fig. 5a). The corresponding decrease in the value of *α* highlights additional compression of the exciton wavefunction in the quantum well due to dielectric confinement, which results in enhanced values of exciton binding energy as compared to merely including the quantum confinement effect (Fig. 5b). The surprisingly good agreement of the expression (2) with experimental results, only using a *γ* correction factor independent of the *n*-value to take into account dielectric effects, will be understood in future studies involving different RPP compositions (Br- and Sn-based 2D perovskites, various organic spacing layers, etc.). We also note that in the simplistic approximation of Hydrogen exciton model, the changes of exciton confinement with perovskite layer thickness can be understood as changes of both the effective dielectric constant value and the exciton Bohr radius as a function of *n*-value (Supplementary Fig. 10).

Applying our model (2) for large *n*-values, we predict that the exciton binding energy in RPPs is larger than room temperature thermal fluctuations (*k*_B_*T*) up to *n* about 20 (perovskite layer thickness of 12.6 nm). This demonstrates the surprising robustness of exciton states in RPPs, in spite of the small spatial extension of the exciton as compared to the perovskite layer thickness. In other words, a more abrupt reduction of the exciton binding energy with increasing perovskite layer thickness was expected given the strong screening of the electron–hole Coulomb interaction reported in 3D perovskites^16^, a situation approached by RPPs with perovskite layer thickness of a few nanometers. Again, this underlines the unusual nature of photo-excited states in RPPs, and to more extent in solution-processed quantum well systems as a template for exploring quasi-2D semiconductors.

In summary, we demonstrate the importance of Coulomb interactions in 2D layered perovskites and experimentally elucidate properties of unexpectedly strongly bound excitons (>120 meV) in RPPs with thickness up to 3.1 nm (corresponding to *n* equals to 5). We propose a generic formulation of the scaling of the exciton binding energy with the perovskite layer thickness from the single layer (*n* equals to 1) to 3D crystals (*n* tends to infinity), further predicting the nature of optical transitions at room temperature to change from excitonic to free carrier like only in RPPs with thickness larger than ~12 nm (*n* about 20). These results mark a fundamental step towards the design of new 2D perovskite-based semiconductor materials for next-generation optoelectronic and photonic technologies such as solar cells, light-emitting diodes, photodetectors, polariton and electrically driven lasers.

## Methods

### RPP crystal synthesis and preparation

The crystal structures of the RPPs, (BA)_2_(MA)_*n*-1_Pb_*n*_I_3*n*+1_, is composed of an anionic layer {(MA)_*n*-1_Pb_*n*_I_3*n*+1_}^2−^, derived from bulk methylammonium lead triiodide perovskites (MAPbI_3_), which is sandwiched between n-butylammonium (BA) spacer cations (Fig. 1a). BA and MA stand for CH_3_(CH_2_)_3_NH_3_ and CH_3_NH_3_, respectively. RPPs with *n* ranging from 1 to 5, corresponding to perovskite layer thickness between 0.641 and 3.139 nm, were synthetized and purified following previously reported method^3–5^. More precisely, the raw crystals were prepared by combining PbO, MACl and BA in appropriate molar ratios in a HI/H_3_PO_2_ solvent mixture. The precursor solutions were prepared with 0.225 M of Pb^2+^ concentration and stirred at room temperature overnight. Phase purity and crystalline quality of each crystal sample was established by monitoring X-ray diffraction (Supplementary Fig. 1). In addition, the small Stokes shifts and relatively sharp linewidths of the exciton resonances (Fig. 3) were another indication of the homogeneity and low disorder in the RPP crystals. Thin RPP crystals were mechanically exfoliated onto either the 3.5 μm core of a single-mode optical fiber for magneto-absorption spectroscopy or transparent quartz substrates for optical absorption, PL, and PLE spectroscopy experiments.

The 3D perovskite MAPbI_3_ (*n* tends to infinity) samples for magneto-absorption spectroscopy were prepared as thin films on transparent substrates using the hot-casting method previously reported^56^.

### Magneto-absorption spectroscopy

A single RPP crystal was affixed over the core of a 3.5 µm diameter single-mode optical fiber to ensure rigid optical alignment of the light path during the magnetic field pulse. The fiber-sample assembly was mounted in a custom optical probe that was fitted in the 4 K bore of a 65 T capacitor-driven pulsed magnet. The sample was in a helium exchange gas environment to ensure thermal anchoring at 4 K. White light from a Xe lamp transmits the sample via the single-mode fiber and was retro-reflected and dispersed in a 300 mm spectrometer with a 300 groove/mm grating. Broadband spectra were recorded every 2.3 ms throughout the magnet pulse. Access to σ^+^ and σ^−^ circular polarization was achieved via a thin-film circular polarizer mounted directly after the sample and by reversing the direction of the magnetic field. Details of the setup can be found elsewhere^57^.

The 3D perovskite MAPbI_3_ thin films were measured in the same manner as described above by placing film sample in front of the core of the optical fiber used for illumination.

### Optical absorption, PL and PLE

Optical spectroscopies were performed with an in-lab-built confocal microscopy system focusing close to the diffraction limit (about 1 μm resolution), a monochromatic laser tunable over the visible and near-infrared spectral ranges. PL spectral responses were obtained through a spectrograph (Spectra-Pro 2300i) and a CCD camera (EMCCD 1024B) yielding an error of less than 2 nm. PL data in the main text were measured for light excitation at 440 nm (if not mentioned otherwise) and the excitation intensity was typically of the order of or below 10^3^ mW cm^−2^. PLE spectra were obtained by measuring the PL integrated intensity while scanning with a 2 nm step the excitation light wavelength at wavelengths smaller than the one of the PL emission peak. Absorption spectra were measured either via a balanced photodiode by tuning the laser excitation wavelength or by detecting the spectral transmission/reflection of the samples exposed to white light. Samples were measured under vacuum (between 10^−5^ and 10^−6^ Torr) and cooled at 4 K if not mentioned otherwise.

### Theory

A momentum space representation of the exciton Green’s function developed previously for classic quantum wells^58^ was used to solve the Bethe–Salpeter equation in the effective mass approximation. This method was adapted for RPPs to include the screening of the electron–hole interaction related to the dielectric confinement and the electron and hole wavefunctions overlap. These latter effects were evaluated at the DFT level. The Wannier–Mott exciton Rydberg states appear as the bound states in the absorption spectrum and can also be determined from the corresponding Schrödinger equation for the two particle wave functions (see details in Supplementary Note 1, 2, and 6).

### Data availability

The data that support the findings of this study are available from the corresponding authors upon reasonable request.

## Electronic supplementary material

Supplementary Information
