# Supplementary material for: Scaling law for excitons in 2D perovskite quantum wells
Source: Nat Commun. 2018 Jun 8;9:2254. doi: 10.1038/s41467-018-04659-x (PMC5993799; doi:10.1038/s41467-018-04659-x)
Supplement: Supplementary file 1 — Supplementary Information [file 41467_2018_4659_MOESM1_ESM.pdf]

SUPPLEMENTARY INFORMATION
for
Scaling law for excitons in 2D perovskite quantum wells

J.-C. Blancon[1]*, A. V. Stier[1], H. Tsai[1,2], W. Nie[1], C. C. Stoumpos[3], B. Traoré[4], L. Pedesseau[5], M. Kepenekian[4], F. Katsutani[6], G. T. Noe[6], J. Kono[2,6,7], S. Tretiak[1], S. A. Crooker[1], C. Katan[4], M. G. Kanatzidis[3,8], J. J. Crochet[1], J. Even[5]* and A. D. Mohite[1,9]*

[1]Los Alamos National Laboratory, Los Alamos, New Mexico 87545, USA.

[2]Department of Materials Science and Nanoengineering, Rice University, Houston, Texas 77005, USA.

[3]Department of Chemistry, Northwestern University, Evanston, Illinois 60208, USA.

[4]Univ Rennes, ENSCR, INSA Rennes, CNRS, ISCR (Institut des Sciences Chimiques de Rennes) - UMR 6226, F-35000 Rennes, France.

[5]Univ Rennes, INSA Rennes, CNRS, Institut FOTON - UMR 6082, F-35000 Rennes, France.

[6]Department of Electrical and Computer Engineering, Rice University, Houston, Texas 77005, USA.

[7]Department of Physics and Astronomy, Rice University, Houston, Texas 77005, USA.

[8]Department of Materials Science and Engineering, Northwestern University, Evanston, Illinois 60208, USA.

[9]Department of Chemical and Biomolecular Engineering, Rice University, Houston, Texas 77005, USA.

*Correspondence to: jblancon@lanl.gov, jacky.even@insa-rennes.fr, adm4@rice.edu

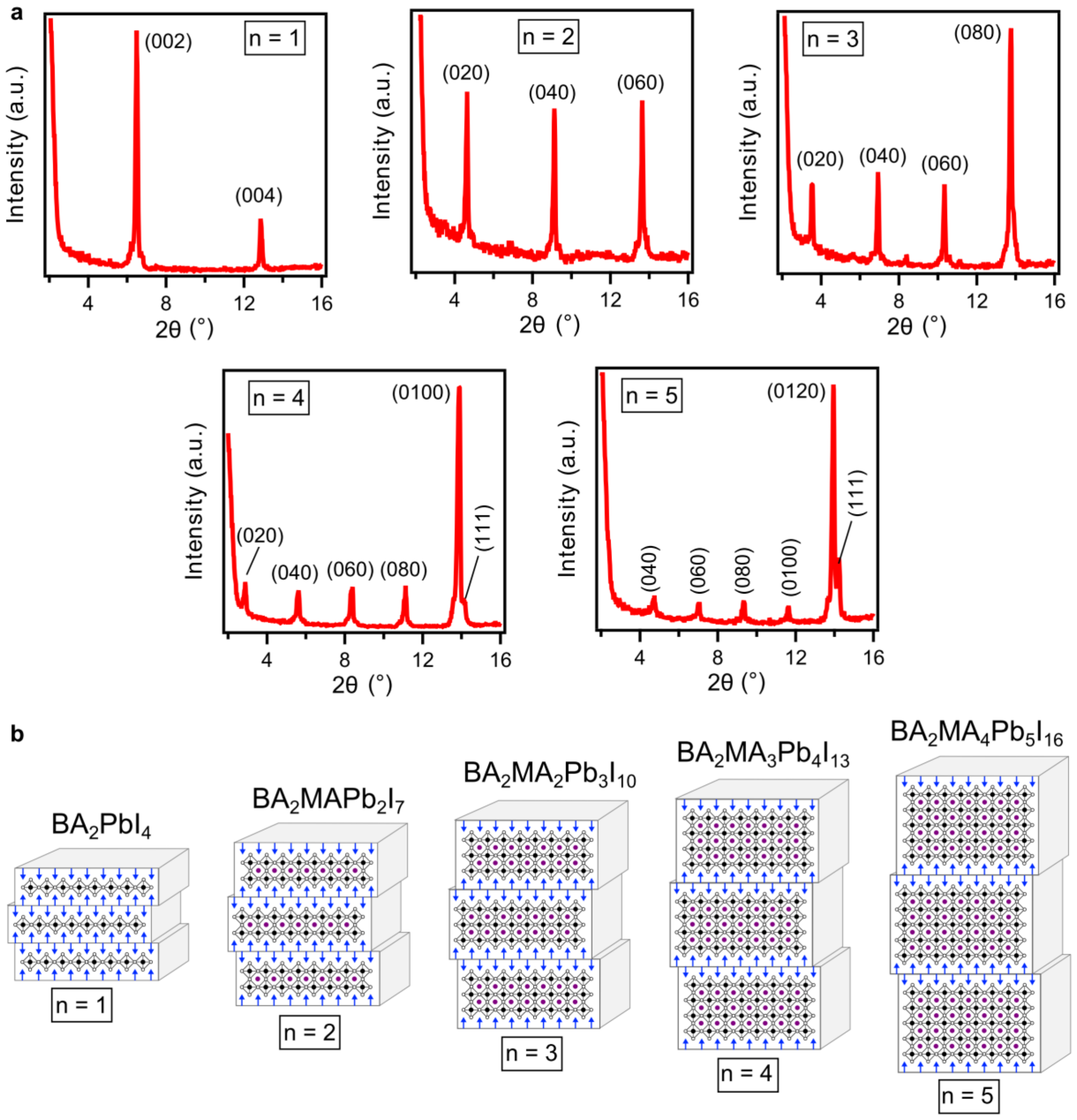

Supplementary Figure 1. Phase purity and crystal structure of RPPs with n=1 to 5. a, X-ray diffraction spectra in the low diffraction angle region (<16°) demonstrating phase purity of each RPP compound. **b,** Corresponding sketch of the crystal structure[1–3] along the stacking axis.

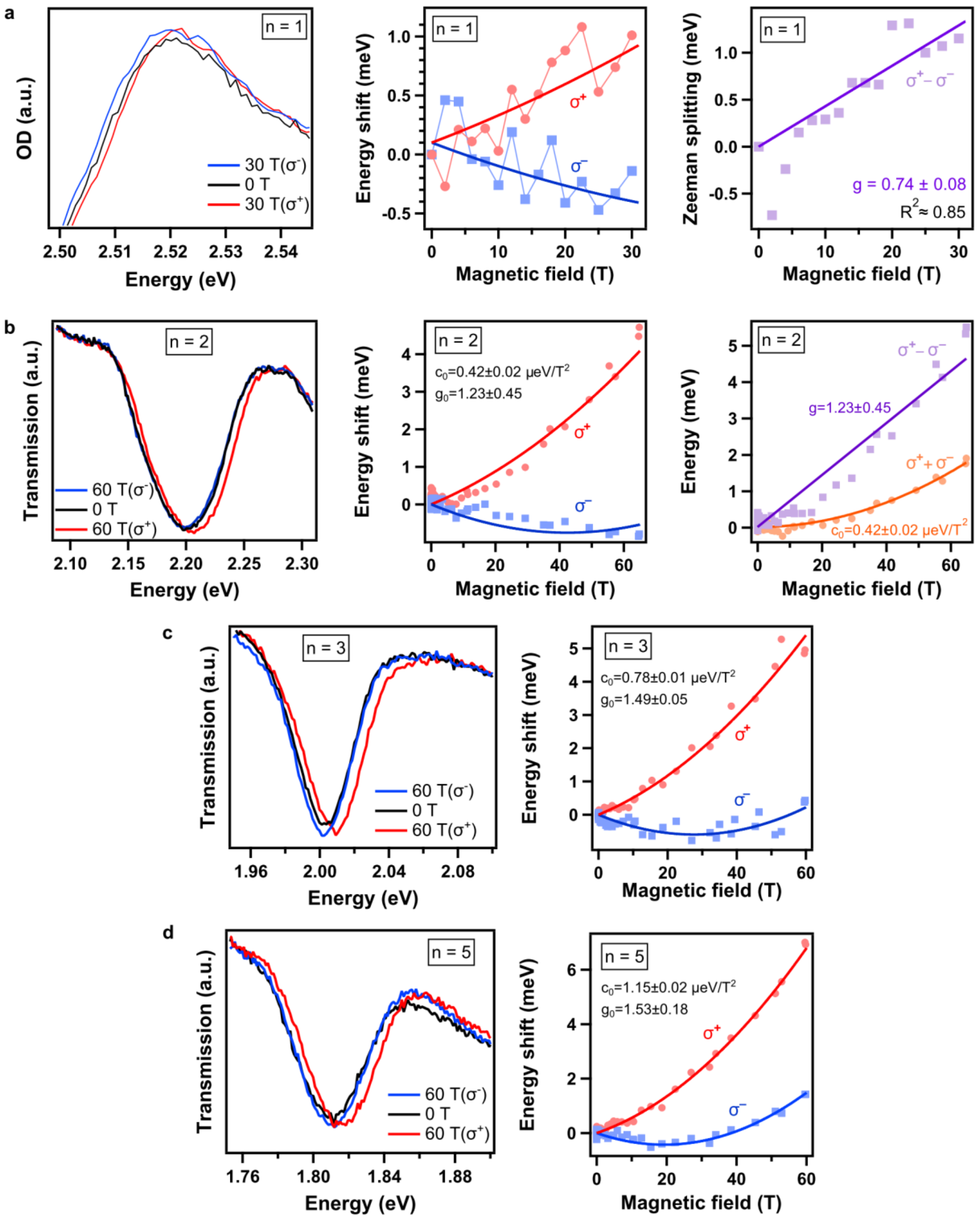

Supplementary Figure 2. Magneto-absorption spectroscopy of RPPs with n=1, 2, 3, 5. The results for RPP n=4 compound are presented in Figure 1. The energy shifts of the exciton ground state (1s) for both right- and left-handed circularly polarization light $\boldsymbol{\sigma}^{\pm}$ were fitted using the classic formula[4] $\boldsymbol{\Delta E} = \pm\frac{1}{2}\boldsymbol{g_0}\boldsymbol{\mu_{\mathrm{B}}}\boldsymbol{B} + \boldsymbol{c_0}\boldsymbol{B^2}$, where the first term stand for the Zeeman splitting ($\boldsymbol{g_0}$ the g-factor in the perovskite plane, $\boldsymbol{\mu_{\mathrm{B}}}$ the Bohr magneton, B the magnetic field) and the second one for the diamagnetic shift ($\boldsymbol{c_0}$ the diamagnetic coefficient). OD stands for optical density.

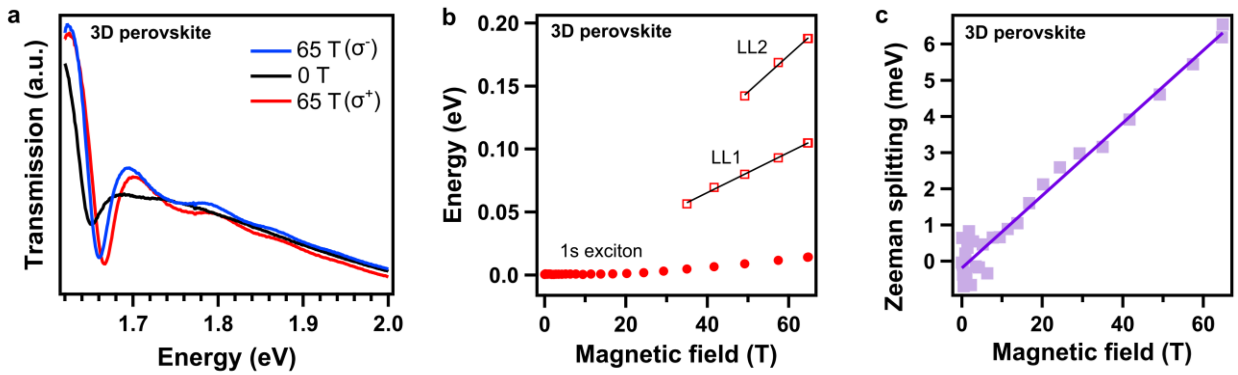

Supplementary Figure 3. Magneto-absorption spectroscopy of 3D perovskite $MAPbI_3$. a, Transmission spectra at 0 T and at the magnetic field extrema 65 T for the two polarization $\sigma^{\pm}$. Under magnetic field we observe both a shift of the peak associated with the exciton ground state (1s) at about 1.65 eV and the appearance of new states at high energy at high magnetic field (ripple-like features above 1.7 eV at ±65 T). The latter features correspond to Landau levels[4,5], and their observation only in 3D perovskite is indicative of a much lower exciton binding energy in 3D perovskite as compared to the RPPs with n=1 to 5. In the high magnetic field region, the evolution of the Landau levels are described by $\Delta E = \left(N_\mathrm{L} + \frac{1}{2}\right)\hbar\omega_\mathrm{c} \pm \frac{1}{2} g_0 \mu_\mathrm{B} B$, with $\omega_\mathrm{c} = eB/\mu$ the cyclotron frequency, N_L (=0, 1, 2, etc.) the Landau quantum number, and the other parameters are defined in the main text. **b,** Corresponding magnetic field dependence of the 1s exciton and the two first Landau levels (noted LL1 and LL2). These data are the average of both polarization $\sigma^{\pm}$ so as they do not include the Zeeman effects, *i.e.*, $\Delta E = \left(N_\mathrm{L} + \frac{1}{2}\right)\hbar\omega_\mathrm{c}$. From the linear fit of the Landau level data we derive the exciton reduced mass of the 3D perovskite: 0.109 m_0 for LL1 and 0.099 for LL2. The average exciton mass μ=0.104 m_0 is identical to the one reported by Miyata *et al.*[5]. In our experiment we did not observe the 2s exciton state as reported by Miyata *et al.*[5], which prevented us from obtaining an exact exciton binding energy in 3D perovskite because the dielectric constant of the system is a priori unknown. Because we obtain experimentally the same effective mass as in ref.[5] in the same 3D perovskite compound, we can assume nearly identical exciton binding energy (16 meV) and effective dielectric constant (ε_eff =9.3). **c,** Zeeman splitting for the 1s exciton yielding a g-factor of g_0=1.7 (the data were fitted by $g_0\mu_\mathrm{B}B$).

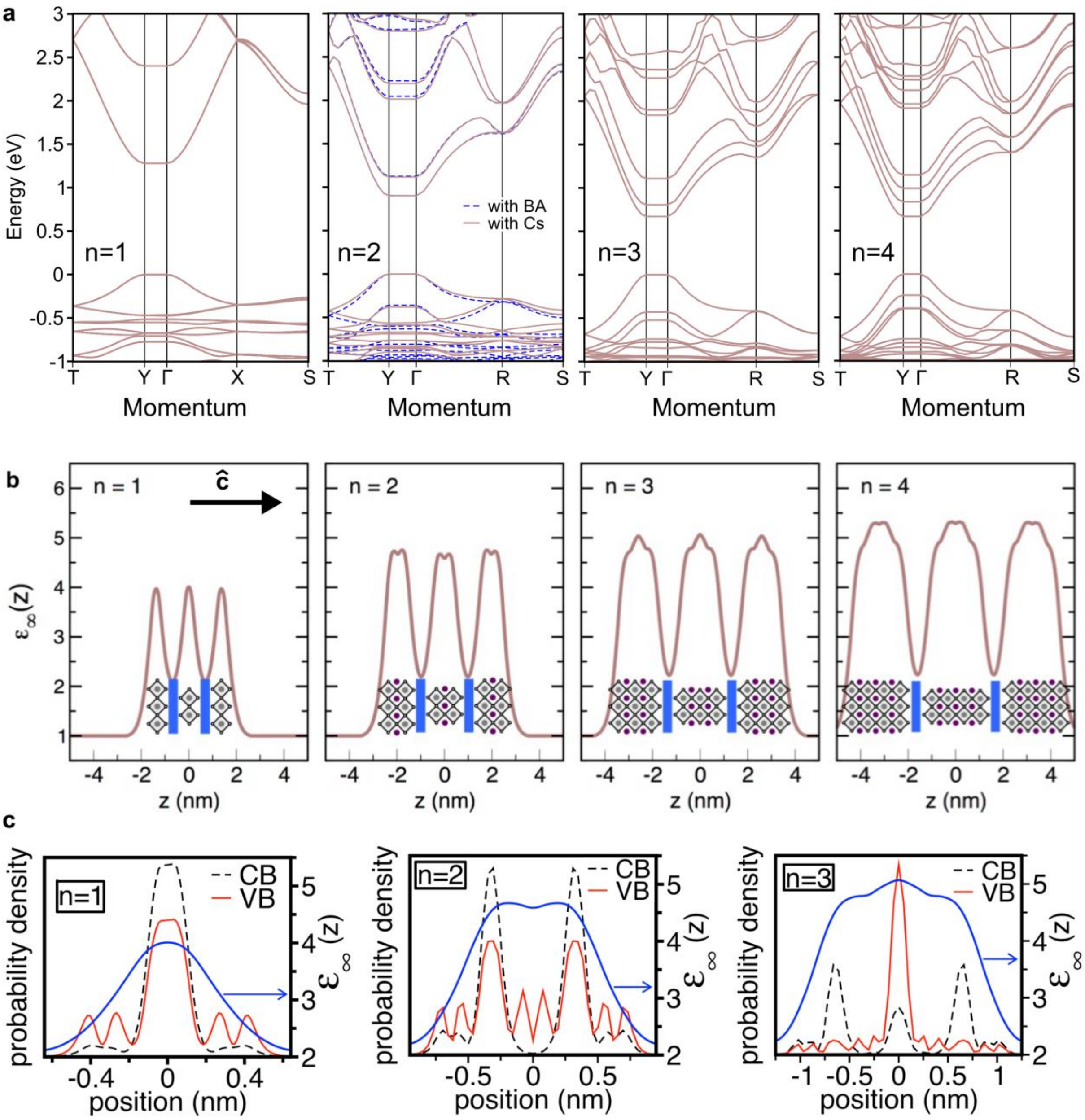

Supplementary Figure 4. DFT computed properties of RPPs with n=1 to 4. a, Computed band structures. Comparison of the band structure computed for the real crystal structure (blue) with that obtained by replacing the organic cations BA with Cs^+ (red) is shown for n=2. **b,** Computed high frequency dielectric constant profiles along the stacking axis ĉ. **c,** Corresponding probability densities of valence (VB, red) and conduction (CB, dashed black lines) bands in a single dielectric quantum well (Figure 2a). For comparison, the dielectric profiles are plotted in blue. Here, the x-axis stands for the position along the stacking axis ĉ centered at the middle of a perovskite layer and spanning a single perovskite layer surrounded by an organic spacer layer on each side. We note that the densities are maximum around the position of the Pb atoms. The dielectric profiles show a strong contrast between the perovskite layer (position 0) and the organic spacer layers (both extrema positions on the x-axis).

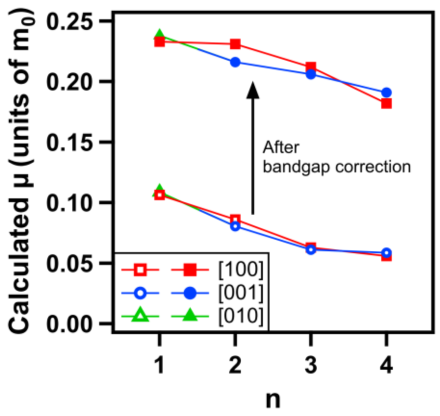

Supplementary Figure 5. DFT computed exciton reduced mass as a function of n, before and after band gap correction. The results are plotted for the [100] in-plane direction and [001] ([010] for the RPP n=1) out-of-plane direction. In order to account for the well-known underestimation of the electronic band gap at the DFT level, the computed single particle effective masses are multiplied by the ratio between the experimental and computed electronic band gaps.

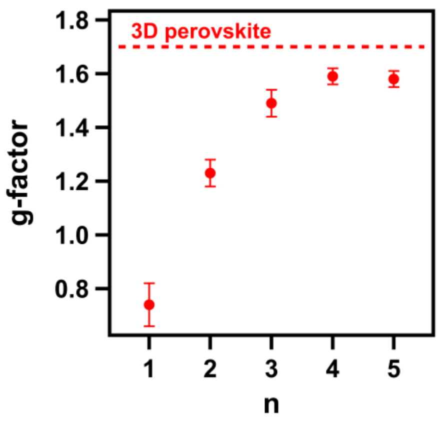

Supplementary Figure 6. Evolution of the g-factor with perovskite layer thickness (n-value).

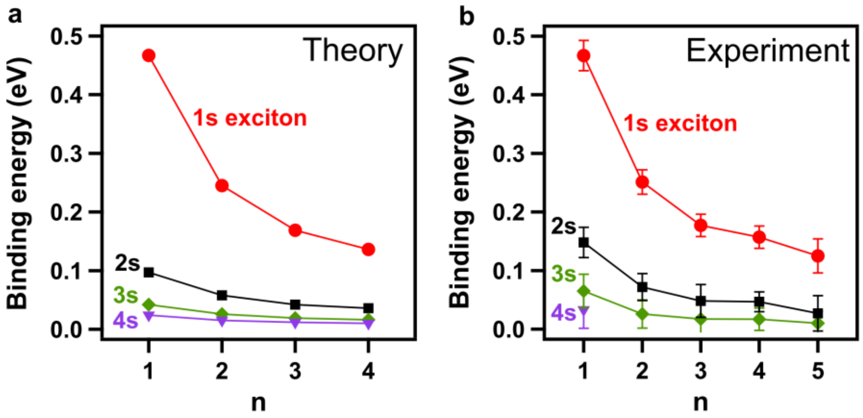

Supplementary Figure 7. a, Calculated and b, measured binding energy of the exciton ground state (1s) and Rydberg series of the excited exciton states (2s, 3s, 4s).

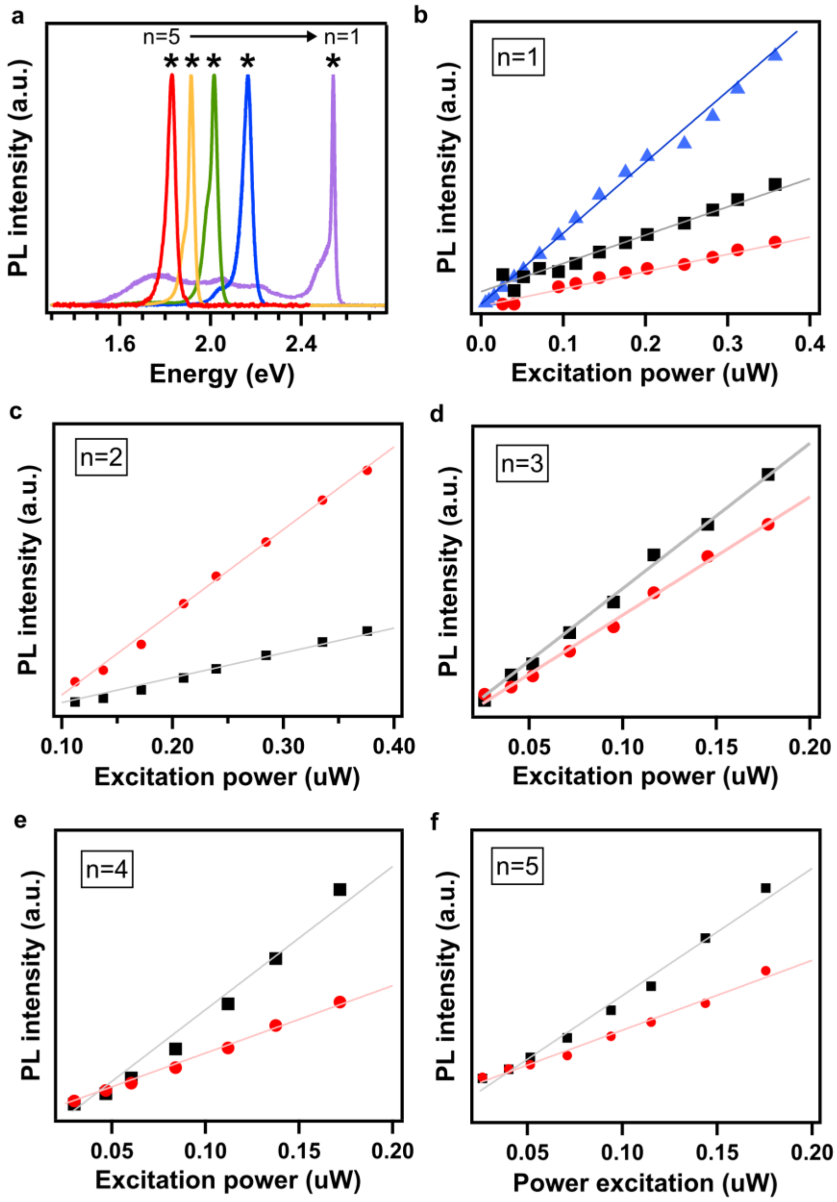

Supplementary Figure 8. Study of the PL spectra features using excitation intensity dependence of the PL at 4K. a. Photoluminescence spectra showing the main exciton ground state peak (*) along with low-energy side features. **b-f.** Excitation power dependence of the main exciton peak (red circles), the low-energy shoulder of the main PL peak (black squares), and the broad PL emission observed in n=1 between 1.5 and 2.4 eV (blue triangles). Excitation was performed at either 440 nm or 480 nm. The integrated signals of all PL features show a nearly linear dependence on the excitation power in the power range used experimentally. These observations suggest that the PL side features have an intrinsic origin, as opposed to excitons bound to defects (sublinear dependence) or bi-excitons (sublinear behavior). Therefore, we hypothesize that the side features are phonon replica and/or self-trapped excitons as suggested in previous reports[6,7], however further studies will be necessary to understand their properties.

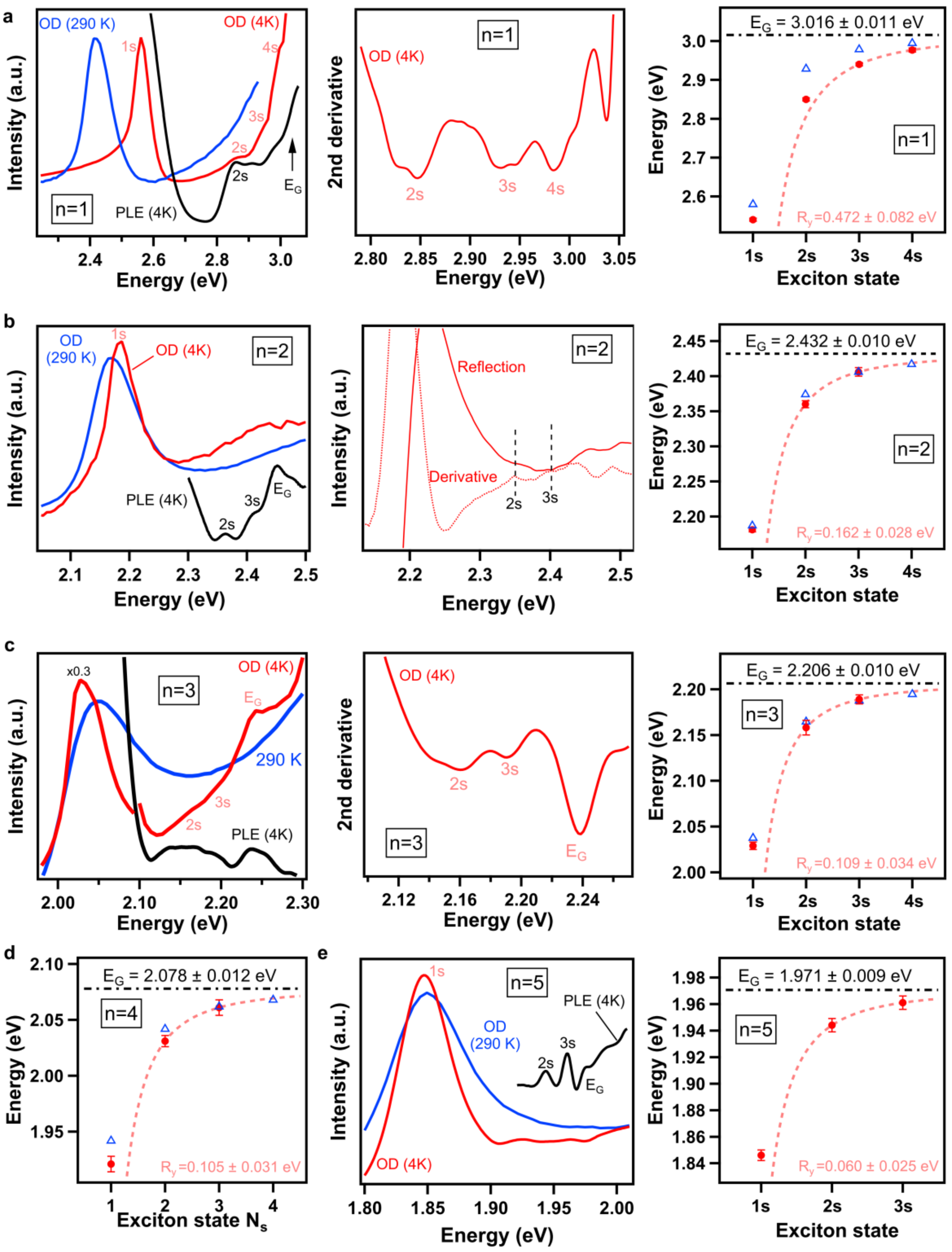

Supplementary Figure 9. Experimental analysis of the exciton Rydberg series in RPPs with n=1 to 5, corresponding to a, to e, respectively. Results follow the method introduced in the main text and in Figure 4. In the plots of the energy versus exciton state the red dots are experimental data derived from absorption and PLE, and the blue triangles correspond to the theoretical results. The dashed lines correspond to the fit of the excited exciton states (2s, 3s, etc.) using the 2D hydrogen model of exciton Rydberg series. R_y is the Rydberg energy.

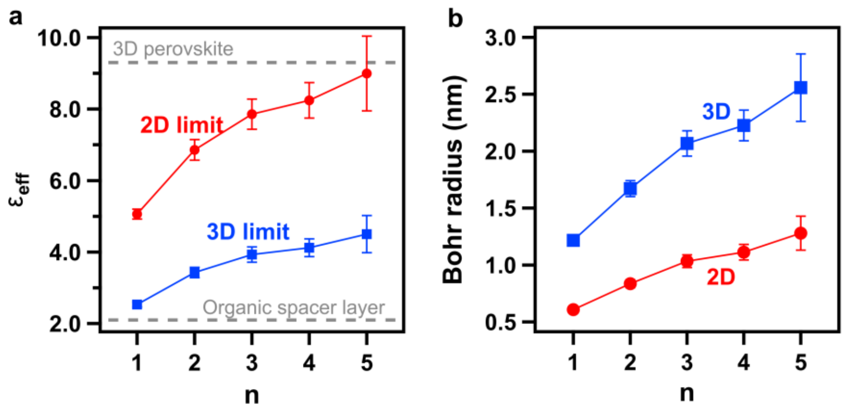

Supplementary Figure 10. Hydrogen exciton model applied to the 1s exciton state in RPPs. a, The effective dielectric constant ε_{eff}, which describes the effective screening of the electron-hole Coulomb interaction, was derived in both the 3D and 2D limiting cases considering only quantum confinement[4], i.e. using the hydrogen model of the exciton $E_{1s} = E_G - R_y$ for 3D and $E_{1s} = E_G - 4R_y$ for 2D, with the Rydberg energy $R_y = (13.6\ \text{eV}) \times \mu/\varepsilon_{eff}^2$. From our interpretation of the experimental and theoretical results, ε_{eff} should adopt a value between the 2D and 3D cases. The RPP n=1 tends to a pure 2D system but with significant contribution of dielectric confinement, and on the other hand, the RPP n=5 tends to deviate from the 2D case. **b,** Corresponding Bohr radius. We emphasize that these results are inexact in nature because the hydrogen exciton model assumed Dirac wavefunctions and does not account for dielectric confinement. This is a first level of approximation to our far more advanced theoretical model, but worth mentioning as a point of comparison of RPPs to other low dimensional systems.

n	RPPs	Perovskite layer thickness (nm)[a]	Exciton reduced mass μ (units of m_0)	Exciton binding energy (meV)
1	$(BA)_2Pb_1I_4$	0.641	0.221	467 ± 26
2	$(BA)_2(MA)Pb_2I_7$	1.255	0.217	251 ± 21
3	$(BA)_2(MA)_2Pb_3I_{10}$	1.892	0.201	177 ± 19
4	$(BA)_2(MA)_3Pb_4I_{13}$	2.511	0.196	157 ± 19
5	$(BA)_2(MA)_4Pb_5I_{16}$	3.139	0.186 (extrapolated)	125 ± 29
∞	$MAPbI_3$ [b]	∞	0.104	16 ± 2

Supplementary Table 1. Summary of exciton properties in RPPs derived from the experimental data. [a]Data from Stoumpos *et al.*[1,3], the organic spacer layer thickness is typically 0.710 nm. [b]From Miyata *et al.*[5].

n	RPPs	Quantum well thickness (nm)	Quantum well dielectric constant ε_w	Spacer layer (barrier) dielectric constant ε_b	Calculated exciton binding energy (meV)
1	$(BA)_2Pb_1I_4$	0.56	4.00	2.10	467
2	$(BA)_2(MA)Pb_2I_7$	1.13	4.64	2.19	245
3	$(BA)_2(MA)_2Pb_3I_{10}$	1.70	4.90	2.22	169
4	$(BA)_2(MA)_3Pb_4I_{13}$	2.28	5.19	2.23	136

Supplementary Table 2. Calculated exciton properties in RPPs and corresponding materials parameters. Details in Supplementary Text 1 and Supplementary Figure 4.

Supplementary Note 1:
Semi-empirical simulation of the Wannier-Mott exciton

The exciton states were modelled using the 3D momentum space representation of the Bethe-Salpeter equation (BSE) for the polarization function P_{eh}, including a statically screened electron-hole (e-h) potential interaction V_{s}, and assuming the effective mass approximation for the electronic dispersions:[8]

$$P_{\mathrm{eh}}(\boldsymbol{k}_{\mathrm{e}},\boldsymbol{k}_{\mathrm{h}},E)=P_{\mathrm{eh}}^{0}(\boldsymbol{k}_{\mathrm{e}},\boldsymbol{k}_{\mathrm{h}},E)-\sum_{\boldsymbol{k}}\sum_{\boldsymbol{k}'}P_{\mathrm{eh}}^{0}(\boldsymbol{k}_{\mathrm{e}},\boldsymbol{k},E)\,V_{\mathrm{s}}(|\boldsymbol{k}-\boldsymbol{k}'|)P_{\mathrm{eh}}^{0}(\boldsymbol{k}',\boldsymbol{k}_{\mathrm{h}},E). \quad (1)$$

In this expression, $\boldsymbol{k}_{\mathrm{e}}$ ($\boldsymbol{k}_{\mathrm{h}}$) is the electron (hole) wavevector in momentum space, E the energy, and the zero-order polarization function in the low-injection regime reads

$$P_{\mathrm{eh}}^{0}(\boldsymbol{k}_{\mathrm{e}},\boldsymbol{k}_{\mathrm{h}},E)=\frac{1}{E-E_{G}-\frac{\hbar^{2}|\boldsymbol{k}_{\mathrm{e}}|^{2}}{2\mu}+i\Gamma}\delta_{\boldsymbol{k}_{\mathrm{e}},\boldsymbol{k}_{\mathrm{h}}}\,, \quad (2)$$

with E_G is the free charge electronic bandgap and Γ a broadening factor.

The potential function describing the Coulomb interaction between the electron and hole forming the exciton states was expressed for the dielectric quantum well in Figure 2a. Although a real space approximate expressions can be used to analyse exciton in 2D materials[9], our model is based on a more general approach which described e-h interaction including dielectric confinement effects in the Fourier space. This method does not require any approximation on the system, and was recently proposed in a different context[10]. Therefore, the partial Fourier transform of the non-local, screened e-h pair interaction potential, in the ($\hat{\boldsymbol{a}}$,$\hat{\mathbf{b}}$) in-plane direction, is given by:[11]

$$V_{\mathrm{s}}(q_{\mathrm{t}})=\frac{-e^{2}}{2\varepsilon_{\mathrm{w}}q_{\mathrm{t}}}\iint_{\frac{-d}{2},\frac{-d}{2}}^{\frac{d}{2},\frac{d}{2}}\rho_{\mathrm{e}}(z_{\mathrm{e}})\rho_{\mathrm{h}}(z_{\mathrm{h}})\big[e^{-q_{\mathrm{t}}|z_{\mathrm{e}}-z_{\mathrm{h}}|}+\Delta\chi\big(e^{-q_{\mathrm{t}}|z_{\mathrm{e}}+z_{\mathrm{h}}-d|}+e^{-q_{\mathrm{t}}|z_{\mathrm{e}}+z_{\mathrm{h}}+d|}\big)$$
$$+\Delta\chi^{2}\big(e^{-q_{\mathrm{t}}|z_{\mathrm{e}}-z_{\mathrm{h}}-2d|}+e^{-q_{\mathrm{t}}|z_{\mathrm{h}}-z_{\mathrm{e}}-2d|}\big)\big]dz_{\mathrm{e}}dz_{\mathrm{h}}, \quad (3)$$

where the difference of electron and hole transverse (in-plane) wavevectors is $q_{\mathrm{t}}=|\boldsymbol{k}_{\mathrm{te}}-\boldsymbol{k}_{\mathrm{th}}|$, $\Delta=(1-\chi^{2}e^{-2q_{\mathrm{t}}d})^{-1}$, and $\chi=\frac{\varepsilon_{\mathrm{w}}-\varepsilon_{\mathrm{b}}}{\varepsilon_{\mathrm{w}}+\varepsilon_{\mathrm{b}}}$. Here, ρ_{e} and ρ_{h} stand for, respectively, the part of the electron and hole probability density profiles along the stacking axis $\hat{\mathbf{c}}$ located inside the dielectric well ($z_{\mathrm{e,h}}$ are real space coordinates along this direction). The leakage of the density profiles in the barrier was also considered and complementary expressions of the interaction potential have been used[11]. This method is equivalent to the real-space computation of the screening effect using the image-charge method[12]. We note that a Gaussian quadrature method was used to integrate the singularity of the e-h interaction in the reciprocal space[13]. The general expression (3) of the e-h interaction potential corresponds to the general Keldysh's potential in real space[14]. Upon several approximations, being $z_{\mathrm{e}}=z_{\mathrm{h}}$, $q_{\mathrm{t}}d\ll 1$, and $\varepsilon_{\mathrm{w}}\gg\varepsilon_{\mathrm{b}}$, the e-h interaction potential used in the

literature in the limit of mono-layer 2D transition metal dichalcogenides[9,15] can be retrieved (see also Supplementary Note 3). However, these approximations are not valid in RPPs and the general expressions need to be used[11].

Supplementary Note 2:
Theoretical solution to the exciton binding energy of the Wannier-Mott exciton

As described in the main text, theoretical solution to the exciton binding energy in RPPs requires the knowledge of *(i)* the electron and hole probability density profiles, *(ii)* the dielectric constant profile along the stacking direction, and *(iii)* the exciton reduced mass.

(i) *Electron and hole probability density profiles*. First, the electron and hole wavefunctions, in RPPs, with in-plane ($\hat{\boldsymbol{a}}$,$\hat{\mathbf{b}}$) isotropic electronic dispersions associated with the transverse wavevector $\boldsymbol{k}_{\mathrm{t}}$, were calculated from[13]:

$$\psi_{\mathrm{e},\boldsymbol{k}_{\mathrm{t}}}(\boldsymbol{r}_{\mathrm{te}}, z_{\mathrm{e}}) = \frac{e^{-i\boldsymbol{k}_{\mathrm{t}}.\boldsymbol{r}_{\mathrm{te}}}}{\sqrt{A}} u_{\mathrm{e},\boldsymbol{k}_{\mathrm{t}}=0}(\boldsymbol{r}_{\mathrm{te}}, z_{\mathrm{e}}), \text{and}$$
$$\psi_{\mathrm{h},\boldsymbol{k}_{\mathrm{t}}}(\boldsymbol{r}_{\mathrm{th}}, z_{\mathrm{h}}) = \frac{e^{-i\boldsymbol{k}_{\mathrm{t}}.\boldsymbol{r}_{\mathrm{th}}}}{\sqrt{A}} u_{\mathrm{h},\boldsymbol{k}_{\mathrm{t}}=0}(\boldsymbol{r}_{\mathrm{th}}, z_{\mathrm{h}}), \quad (4)$$

where $u_{\mathrm{e},\boldsymbol{k}_{\mathrm{t}}=0}(\boldsymbol{r}_{\mathrm{te}}, z_{\mathrm{e}})$ and $u_{\mathrm{h},\boldsymbol{k}_{\mathrm{t}}=0}(\boldsymbol{r}_{\mathrm{th}}, z_{\mathrm{h}})$ are the ground-state, complex spinor Bloch functions at the Brillouin zone center, for the electron and hole, respectively, and A is the surface introduced for normalization purpose. Then the electron and hole probability density profiles along the stacking axis, corresponding to the envelope functions in classic quantum wells[12,13], were derived by averaging in-plane the Bloch functions:

$$\rho_{\mathrm{e}}(z_{\mathrm{e}}) = \iint \left|u_{\mathrm{e},\boldsymbol{k}_{\mathrm{t}}=0}\right|^2 d^2\boldsymbol{r}_{\mathrm{te}} \text{ and } \rho_{\mathrm{h}}(z_{\mathrm{h}}) = \iint \left|u_{\mathrm{h},\boldsymbol{k}_{\mathrm{t}}=0}\right|^2 d^2\boldsymbol{r}_{\mathrm{th}}. \quad (5)$$

In practice, the spinor Bloch functions were calculated only for the highest valence band and lowest conduction band without loss of information and negligible error on the results.

The charge density profiles (Figure 2c and Supplementary Figure 4c) were obtained from the expressions (4) and (5) using the wavefunctions calculated by DFT including spin-orbit coupling. This approach was necessary because methods using effective mass approximation associated with envelope functions along the stacking axis have shown to be unsuitable for predicting the electronic state energies and wavefunctions in RPPs[16]. Precisely, non-physical superlattice effects are predicted by such methods whereas DFT results exhibit flat dispersions along the stacking axis (Figure 2b, Supplementary Figure 4a, and refs.[3,17]). The fundamental reason explaining this non-conventional behavior is that in RPPs the quantum wells (perovskite layers) and surrounding well barriers (organic spacing layers) do not share common bulk basis Bloch functions, unlike classic semiconductor heterostructures[16]. The density profiles show that both the electron and hole have maximum probability density at the Pb-atom locations, and that the conduction and valence band wavefunctions do not overlap perfectly. Convolution of the electron and hole densities along the stacking axis demonstrated that the distribution of e-h distances within the exciton ground state has slightly larger spatial expansion for the RPPs n=3,4 than their n=1,2 counterparts. However, the electron and hole densities ($\rho_e(z_e)$ and

$\rho_h(z_h)$) have little leakage outside the dielectric quantum well, thereby confirming that the RPPs can be considered as almost infinite quantum wells[16].

(ii) *Dielectric constant profiles along the stacking direction*. Second, the dielectric constant profiles of the quantum well systems were obtained from the DFT calculations (Figure 2c, Supplementary Figure 4b, and Supplementary Table 2) using a methodology developed and reported previously[18–20]. Nevertheless, we also checked that an abrupt dielectric interface approximation does not affect significantly the result for the exciton binding energy, as compared to the DFT-calculated dielectric profiles or considering a more realistic trapezoidal shape. In the latter case, the potential response to a point charge, in the linearly graded dielectric transition region between the perovskite quantum-well and the organic spacer layer, can be computed analytically with modified Bessel functions (I_n, K_n) instead of exponential functions[21]:

$$V_s(q_t) = aI_0\left(\frac{q_t\varepsilon(z)}{|p|}\right) + bK_0\left(\frac{q_t\varepsilon(z)}{|p|}\right),$$

where $\varepsilon(z)$ is the linear variation of the dielectric constant, p its slope and a, b two constants determined by continuity conditions.

(iii) *Exciton reduced mass*. Finally, the exciton reduced mass for each RPP was estimated such that the diamagnetic shift derived from our model matches the experimental values from magneto-absorption (Figure 1d,e). This approach is justified because it has been shown earlier that effective masses computed for hybrid perovskites strongly depend on the accuracy of the theoretical model used[22], and that reliable calculations of effective masses can be obtained only by a self-consistent implementation of both relativistic (SOC) and many-body effects (GW)[23]. More precisely, effective masses strongly depend on the accuracy of the DFT approach, leading to a direct relation between predicted band gaps and effective masses[24]. From the computational point of view, such accuracy of theory, which has already been challenging for small size unit cells in organic-inorganic halide perovskites (as $MAPbI_3$), is out of reach for crystal structures as large as the ones of RPPs. Moreover, the influence of thermal effects on the effective masses cannot be captured by DFT.

Supplementary Note 3:
Examples of approximated cases of the screened electron-hole interaction potential.

- For a *strict screened 2D case* (electron and hole densities approximated by delta functions along the direction of layer stacking, *i.e.* $z_{\mathrm{e}} = z_{\mathrm{h}}$ and $\rho_{\mathrm{e}}(z_{\mathrm{e}}) = \delta_{z_{\mathrm{e}}}$ and $\rho_{\mathrm{h}}(z_{\mathrm{h}}) = \delta_{z_{\mathrm{h}}}$), including dielectric confinement effect, the e-h interaction potential reads

$$V_s(q_t) = \frac{-e^2}{2\varepsilon_w q_t}\frac{1+\chi e^{-q_t d}}{1-\chi e^{-q_t d}}$$

It is equivalent to an effective 2D dielectric function of the form:

$$\varepsilon_{2D}(q_t) = \varepsilon_w \frac{1-\chi e^{-q_t d}}{1+\chi e^{-q_t d}}$$

This case leads to a strong enhancement of the e-h interaction and was analysed initially by Keldysh[14] yielding approximate expressions of the potential.

In the approximation of long range electron-hole interaction (small in-plane wavevectors) and quantum wells with small thickness ($q_{\mathrm{t}} d \ll 1$), a linear form of the effective 2D dielectric function is obtained

$$\varepsilon_{2D}(q_t) = \varepsilon_b + \frac{(\varepsilon_w^2 - \varepsilon_b^2)}{2\varepsilon_w} q_t d,$$

which can be further approximated[14] in the case of $\varepsilon_{\mathrm{w}} \gg \varepsilon_{\mathrm{b}}$ by

$$\varepsilon_{2D}(q_t) = \varepsilon_b + \frac{\varepsilon_w q_t d}{2}.$$

A similar formula can be obtained from *ab initio* approaches for Van der Waals heterostructures in the screened 2D limit introducing a 2D polarizability α for a monolayer in vacuum[10]

$$\varepsilon_{2D}(q_t) = 1 + \frac{\alpha q_t}{2}$$

- For a *pure quantum confinement* effect ($\varepsilon_w = \varepsilon_b, \chi = 0$), the screened e-h interaction reduces to[32]:

$$V_s(q_t) = \frac{-e^2}{2\varepsilon_w q_t}\iint \rho_e(z_e)\rho_h(z_h) e^{-q_t|z_e - z_h|} dz_e dz_h.$$

- For a *pure quantum confinement* effect ($\varepsilon_w = \varepsilon_b$), and in the *strict 2D case* (electron and hole densities approximated by delta functions along the direction of layer stacking, *i.e.* $z_{\mathrm{e}} = z_{\mathrm{h}}$ and $\rho_{\mathrm{e}}(z_{\mathrm{e}}) = \delta_{z_{\mathrm{e}}}$ and $\rho_{\mathrm{h}}(z_{\mathrm{h}}) = \delta_{z_{\mathrm{h}}}$), the e-h interaction reads:

  $$V_s(q_t) = \frac{-e^2}{2\varepsilon_w q_t}$$

  This 2D limit corresponds to a binding energy of the exciton of $4R_y$, where $R_y = \frac{\mu e^4}{2\hbar^2(4\pi\varepsilon_w)^2}$ is the Rydberg energy of the system.

Supplementary Note 4:
Comparison to exciton modelling in Van der Waals heterostructures

Detailed analyses of the exciton binding energy have been proposed recently in the literature for Van de Waals heterostructures such as hBN and MoS_2.[25,26] A full ab initio solution of the BSE was compared to a Mott-Wannier model including a pure 2D screened Coulomb interaction[10] or a new quasi-2D screened Coulomb interaction resulting from an average over step-like representations or actual distributions of the density. It shows that screened 2D or quasi-2D approaches are both in good agreement with a full ab initio solution of the BSE for monolayered Van der Waals heterostructures. By comparison, full ab initio modelling of the BSE for the RPP n=1 case is not possible with the available computational resources. We nevertheless conclude in the present study that screened 2D approximations breaks down due the spatial extension of the density profiles. The method used in the present work is thus more closely related to the quasi-2D approach of Latini *et al.*[25] where actual density profiles can be used to compute the screened electron-hole interaction in reciprocal space. However, the effect of the dielectric mismatch is introduced in the work of Latini *et al.*[25] for Van der Waals heterostructures through a proper 2D transformation of the 3D bulk dielectric, accounting for local field effects. In the present work due to the absence of a 3D reference system, a direct computation of the dielectric profile was performed for each RPP phase.

In Van der Waals heterostructures, the crossover from a 2D monolayer to a 3D bulk crystal can be studied theoretically by increasing the spacing between the layers[10]. It allows computing the 2D polarizability in the 3D bulk reference crystal. For the 3D compound ($MAPbI_3$) corresponding to the RPP phases, covalent bonds are present along the stacking axis. On the other hand, dangling bonds are present in the RPP n=1 structure along the same axis. The $MAPbI_3$ crystal can thus not be regarded as a simple assembly of RPP monolayers, *i.e.* as a simple reference system to evaluate dielectric or quantum confinement effects[16].

Supplementary Note 5:
Computation of the diamagnetic shift.

The diamagnetic shift in the Faraday configuration was computed from $c_0 = \frac{e^2|\boldsymbol{r}_{te}-\boldsymbol{r}_{th}|^2}{8\mu}$, and by numerically averaging the distance between the in-plane ($\hat{\boldsymbol{a}}$,$\hat{\mathbf{b}}$) electron ($\boldsymbol{r}_{te}$) and hole ($\boldsymbol{r}_{th}$) positions.

For information, in the case of a strictly 2D system and for only quantum confinement effects, the analytic expression of the diamagnetic shift $c_0 = \frac{3}{8}\frac{e^2 a_{\mathrm{B}}^2}{8\mu}$ matches the numerical result (a_{B} the exciton Bohr radius).

Supplementary Note 6:
DFT computations of 2D RPPs

Electronic band dispersions and spinor Bloch functions were computed within the Density Functional Theory (DFT)[27,28] as implemented in the ABINIT package[29]. Experimental crystal structures were used, namely the crystal structure recorded at 293K with Pbca space group for n=1,[30] and those reported in Ref. 3 having Cmcm space group for n = 2 and 4 and Cmca for n = 3. Band dispersions and the spinor Bloch functions with the organic cations replaced by Cs^+ were computed using the revPBE gradient correction for exchange-correlation[31] and PAW datasets[32] for Pb, I, Cs, N, C and H as pseudopotentials. The electronic wavefunctions were expanded onto a plane-wave basis set with an energy cutoff of 517 eV. The following Monkhorst-Pack grids for reciprocal space integration were used: 4x4x1 for n=1 and 2x2x4 for n=2, 3 and 4. Spin orbit coupling interaction was taken into account in our model given its significant role in lead-iodide perovskites[33,34]. The computational burden was reduced by replacing the organic cations (BA) by Cs^+ atoms; this method[22,33,34] was shown to have minor impact on the electronic bandstructure of RPPs. DFT results are presented in Figure 2 and Supplementary Figure 4.

High-frequency dielectric constant profiles were computed according to a methodology described elsewhere[18–20], using the DFT implementation of the SIESTA package[35] with a basis set of finite-range of numerical atomic orbitals. We used the generalized gradient approximation with PBE functional[36] to describe the exchange-correlation term. Norm-conserving Troullier-Martins pseudopotentials were used for each atomic species to account for the core electrons[37]. Here, $1s^1$, $2s^2 2p^2$, $2s^2 2p^3$, $5s^2 5p^5$, $5d^{10} 6s^2 6p^2$ were used as valence electrons for H, C, N, I, and Pb, respectively. Polarized Double-Zeta (DZP) basis set with an energy shift of 50 meV were used for the calculations. For the real space mesh grids, energy cutoffs of 200 Rydberg were used. Slabs based on the RPPs were constructed and an electric field of 0.01 eV/A was applied along stacking direction with the relaxation of the sole electron density. The Brillouin zone was sampled with 4x4x1 Monckhorst-Pack grids. To obtain the electronic density of the slabs for the dielectric constant computations, a nanoscale averaging along the stacking axis (c-axis) was performed[38].

Supplementary References

1. Stoumpos, C. C. *et al.* Ruddlesden–Popper hybrid lead iodide perovskite 2D homologous semiconductors. *Chem. Mater.* **28,** 2852–2867 (2016).
2. Cao, D. H., Stoumpos, C. C., Farha, O. K., Hupp, J. T. & Kanatzidis, M. G. 2D homologous perovskites as light-absorbing materials for solar cell applications. *J. Am. Chem. Soc.* **137,** 7843–7850 (2015).
3. Stoumpos, C. C. *et al.* High members of the 2D Ruddlesden-Popper halide perovskites: synthesis, optical properties, and solar cells of $(CH_3(CH_2)_3NH_3)_2(CH_3NH_3)_4Pb_5I_{16}$. *Chem* **2,** 427–440 (2017).
4. Klingshirn, P. C. F. *Semiconductor Optics*. (Springer Berlin Heidelberg, 2012).
5. Miyata, A. *et al.* Direct measurement of the exciton binding energy and effective masses for charge carriers in organic-inorganic tri-halide perovskites. *Nat. Phys.* **11,** 582–587 (2015).
6. Gauthron, K. *et al.* Optical spectroscopy of two-dimensional layered $(C_6H_5C_2H_4\text{-}NH_3)_2\text{-}PbI_4$ perovskite. *Opt. Express* **18,** 5912–5919 (2010).
7. Dohner, E. R., Jaffe, A., Bradshaw, L. R. & Karunadasa, H. I. Intrinsic white-light emission from layered hybrid perovskites. *J. Am. Chem. Soc.* **136,** 13154–13157 (2014).
8. Schmitt-Rink, S., Löwenau, J. & Haug, H. Theory of absorption and refraction of direct-gap semiconductors with arbitrary free-carrier concentrations. *Z. Für Phys. B Condens. Matter* **47,** 13–17 (1982).
9. Chernikov, A. *et al.* Exciton binding energy and nonhydrogenic Rydberg series in monolayer WS_2. *Phys. Rev. Lett.* **113,** 076802 (2014).
10. Cudazzo, P., Tokatly, I. V. & Rubio, A. Dielectric screening in two-dimensional insulators: Implications for excitonic and impurity states in graphane. *Phys. Rev. B* **84,** 085406 (2011).
11. Barrera, R. G., Guzmán, O. & Balaguer, B. Point charge in a three-dielectric medium with planar interfaces. *Am. J. Phys.* **46,** 1172–1179 (1978).
12. Kumagai, M. & Takagahara, T. Excitonic and nonlinear-optical properties of dielectric quantum-well structures. *Phys. Rev. B* **40,** 12359–12381 (1989).
13. Chuang, S.-L., Schmitt-Rink, S., Miller, D. A. B. & Chemla, D. S. Exciton Green's-function approach to optical absorption in a quantum well with an applied electric field. *Phys. Rev. B* **43,** 1500–1509 (1991).
14. Keldysh, L. V. Coulomb interaction in thin semiconductor and semimetal films. *Sov. J. Exp. Theor. Phys. Lett.* **29,** 658 (1979).
15. Berkelbach, T. C., Hybertsen, M. S. & Reichman, D. R. Theory of neutral and charged excitons in monolayer transition metal dichalcogenides. *Phys. Rev. B* **88,** 045318 (2013).

16. Even, J., Pedesseau, L. & Katan, C. Understanding quantum confinement of charge carriers in layered 2D Hybrid Perovskites. *ChemPhysChem* **15,** 3733–3741 (2014).

17. Tsai, H. *et al.* High-efficiency two-dimensional Ruddlesden–Popper perovskite solar cells. *Nature* **536,** 312–316 (2016).

18. Tanaka, K. *et al.* Image charge effect on two-dimensional excitons in an inorganic-organic quantum-well crystal. *Phys. Rev. B* **71,** 045312 (2005).

19. Even, J., Pedesseau, L. & Kepenekian, M. Electronic surface states and dielectric self-energy profiles in colloidal nanoscale platelets of CdSe. *Phys. Chem. Chem. Phys.* **16,** 25182–25190 (2014).

20. Sapori, D., Kepenekian, M., Pedesseau, L., Katan, C. & Even, J. Quantum confinement and dielectric profiles of colloidal nanoplatelets of halide inorganic and hybrid organic–inorganic perovskites. *Nanoscale* **8,** 6369–6378 (2016).

21. Stern, F. Image potential near a gradual interface between two dielectrics. *Phys. Rev. B* **17,** 5009–5015 (1978).

22. Even, J., Pedesseau, L., Jancu, J.-M. & Katan, C. DFT and k · p modelling of the phase transitions of lead and tin halide perovskites for photovoltaic cells. *Phys. Status Solidi RRL – Rapid Res. Lett.* **8,** 31–35 (2014).

23. Filip, M. R., Verdi, C. & Giustino, F. GW band structures and carrier effective masses of $CH_3NH_3PbI_3$ and hypothetical perovskites of the type $APbI_3$: A = NH_4, PH_4, AsH_4 and SbH_4. *J. Phys. Chem. C* (2015). doi:10.1021/acs.jpcc.5b07891

24. van Schilfgaarde, M., Kotani, T. & Faleev, S. Quasiparticle self-consistent GW theory. *Phys. Rev. Lett.* **96,** 226402 (2006).

25. Latini, S., Olsen, T. & Thygesen, K. S. Excitons in van der Waals heterostructures: The important role of dielectric screening. *Phys. Rev. B* **92,** 245123 (2015).

26. Olsen, T., Latini, S., Rasmussen, F. & Thygesen, K. S. Simple screened hydrogen model of excitons in two-dimensional materials. *Phys. Rev. Lett.* **116,** 056401 (2016).

27. Hohenberg, P. & Kohn, W. Inhomogeneous electron gas. *Phys. Rev.* **136,** B864–B871 (1964).

28. Kohn, W. & Sham, L. J. Self-consistent equations including exchange and correlation effects. *Phys. Rev.* **140,** A1133–A1138 (1965).

29. Gonze, X. *et al.* ABINIT: First-principles approach to material and nanosystem properties. *Comput. Phys. Commun.* **180,** 2582–2615 (2009).

30. Billing, D. G. & Lemmerer, A. Synthesis, characterization and phase transitions of the inorganic–organic layered perovskite-type hybrids $(C_nH_{2n+1}NH_3)_2PbI_4]$ (n = 12, 14, 16 and 18). *New J. Chem.* **32,** 1736–1746 (2008).

31. Zhang, Y. & Yang, W. Comment on `Generalized gradient approximation made simple'. *Phys.Rev.Lett.* **80,** 890–890 (1998).
32. Jallet, H., Torrent, M. & Holzwarth, N. Generation of projector augmented-wave atomic data: a 71 element validated table in the XML format. *Comput Phys Commun* **185,** 1246–1254 (2014).
33. Even, J., Pedesseau, L., Dupertuis, M.-A., Jancu, J.-M. & Katan, C. Electronic model for self-assembled hybrid organic/perovskite semiconductors: Reverse band edge electronic states ordering and spin-orbit coupling. *Phys. Rev. B* **86,** 205301 (2012).
34. Even, J., Pedesseau, L., Jancu, J.-M. & Katan, C. Importance of spin–orbit coupling in hybrid organic/inorganic perovskites for photovoltaic applications. *J. Phys. Chem. Lett.* **4,** 2999–3005 (2013).
35. Soler, J. M. *et al.* The SIESTA method for ab initio order- N materials simulation. *J. Phys. Condens. Matter* **14,** 2745 (2002).
36. Perdew, J. P., Burke, K. & Ernzerhof, M. Generalized gradient approximation made simple. *Phys. Rev. Lett.* **77,** 3865–3868 (1996).
37. Troullier, N. & Martins, J. L. Efficient pseudopotentials for plane-wave calculations. *Phys. Rev. B* **43,** 1993–2006 (1991).
38. Junquera, J., Cohen, M. H. & Rabe, K. M. Nanoscale smoothing and the analysis of interfacial charge and dipolar densities. *J. Phys. Condens. Matter* **19,** 213203 (2007).
